# BAY61-3606 Affects the Viability of Colon Cancer Cells in a Genotype-Directed Manner

**DOI:** 10.1371/journal.pone.0041343

**Published:** 2012-07-18

**Authors:** Ken S. Lau, Tinghu Zhang, Krystle R. Kendall, Douglas Lauffenburger, Nathanael S. Gray, Kevin M. Haigis

**Affiliations:** 1 Molecular Pathology Unit, Center for Cancer Research and Center for Systems Biology, Massachusetts General Hospital, Charlestown, Massachusetts, United States of America; 2 Department of Biological Engineering and Koch Institute for Integrative Cancer Research, Massachusetts Institute of Technology, Cambridge, Massachusetts, United States of America; 3 Department of Cancer Biology, Dana Farber Cancer Institute, Boston, Massachusetts, United States of America; University of North Carolina, United States of America

## Abstract

**Background:**

K-RAS mutation poses a particularly difficult problem for cancer therapy. Activating mutations in K-RAS are common in cancers of the lung, pancreas, and colon and are associated with poor response to therapy. As such, targeted therapies that abrogate K-RAS-induced oncogenicity would be of tremendous value.

**Methods:**

We searched for small molecule kinase inhibitors that preferentially affect the growth of colorectal cancer cells expressing mutant K-RAS. The mechanism of action of one inhibitor was explored using chemical and genetic approaches.

**Results:**

We identified BAY61-3606 as an inhibitor of proliferation in colorectal cancer cells expressing mutant forms of K-RAS, but not in isogenic cells expressing wild-type K-RAS. In addition to its anti-proliferative effects in mutant cells, BAY61-3606 exhibited a distinct biological property in wild-type cells in that it conferred sensitivity to inhibition of RAF. In this context, BAY61-3606 acted by inhibiting MAP4K2 (GCK), which normally activates NFκβ signaling in wild-type cells in response to inhibition of RAF. As a result of MAP4K2 inhibition, wild-type cells became sensitive to AZ-628, a RAF inhibitor, when also treated with BAY61-3606.

**Conclusions:**

These studies indicate that BAY61-3606 exerts distinct biological activities in different genetic contexts.

## Introduction

RAS family GTPases act as binary switches that undergo a conformational change upon binding to GTP, allowing them to engage a host of downstream signaling effectors including RAF, PI3K, and RALGDS [1]. The intrinsic GTPase activity of RAS hydrolyzes GTP to GDP, with the help of GTPase activating protein (GAP) cofactors, to inactivate its signaling capability. Missense mutations in codons 12, 13, 61, or 146 are common in cancer and are associated with resistance to GAP activity, allowing RAS to persist in the activated, GTP-bound state. Activating K-RAS mutations occur in approximately 15% of all cancers (making it one of the most commonly mutated oncogenes), but are particularly common in the most lethal forms of cancer, such as those arising in the biliary tract, colon, lung, and pancreas [2]. In colorectal cancer, for example, K-RAS is mutated in nearly 40% of cases [2]. Importantly, tumors with K-RAS mutations are especially refractory to conventional and targeted therapies and are usually associated with poor prognosis [3]–[5].

The main challenge of counteracting the oncogenic effects of activated K-RAS is the inability to directly inhibit the mutant protein. Because the signaling properties of K-RAS are enhanced via inactivation of its GTPase activity, direct pharmacologic inhibition of RAS is not a viable therapeutic strategy. An alternate strategy to counteract mutant K-RAS is to inhibit its downstream effectors, for example, the RAF-MEK-ERK (MAPK) pathway. MEK inhibitors have received attention due their allosteric mechanism of action, which confers extreme specificity, and their demonstrated efficacy in melanomas and colon cancers expressing activated B-RAF [6], [7]. MEK inhibitors perform poorly in cancers expressing mutant K-RAS, however, perhaps due to secondary mutations that affect response or the existence of MEK-independent signaling downstream of RAF [8], [9]. Given the presumptiveness of these scenarios, it is clear that a better understanding of how the K-RAS signaling network operates in cancer is needed to develop novel therapies.

In recent years, large-scale functional genomic approaches have been employed to discover kinase targets that when knocked down are “synthetic lethal” with mutant RAS. Potential therapeutic targets that have been identified include TBK1 [10], STK33 [11], CDK4 [12], and PLK1 [13], although it remains to be seen whether any of these represent *bona fide* therapeutic targets for K-RAS mutant cancers. Whereas understanding the mechanisms by which K-RAS signals through these targets is central to the design of effective drugs, a less studied, and often overlooked, question is why wild-type cells, which also express these targets, tolerate loss of function of these enzymes. This issue is equally important for drug design because the advantage of targeted therapies (over conventional chemotherapies) is their potential selectivity for malignant cells.

In this study, we have characterized the activity of BAY61-3606 in the context of colorectal cancer, providing insight into (1) potential therapeutic targets for cancers expressing mutant K-RAS and (2) pathways that regulate the response of non-mutant cells to targeted inhibitors. BAY61-3606 was originally identified as an orally available, ATP-competitive inhibitor of Spleen Tyrosine Kinase (SYK) [14]. Since SYK plays an active role in inflammatory response, BAY61-3606 has mainly been used for studying immune cell function. For example, BAY61-3606 suppresses antigen-induced airway inflammation in rats and B cell migration in mice [14], [15]. While all of the effects of BAY61-3606 in immune cells are linked to its ability to inhibit SYK, it is unknown whether BAY61-3606 has alternate targets of biological relevance in other cellular contexts. In this study, we have characterized two SYK-independent activities associated with BAY61-3606 in colorectal cancer cells.

## Methods

### Cell lines, knockdowns, and drug treatments

All colon cancer cell lines were maintained in DMEM supplemented with penicillin (100 units/mL), streptomycin (100 μg/mL), and 10% fetal bovine serum (FBS). The rectal cancer cell line (Car1) was maintained in DMEM/F12 supplemented with penicillin (100 units/mL)/streptomycin (50 μg/mL), and 5% FBS. Knockdowns were achieved with pSICOR or pLKO lentiviral vectors [16]. The target sequences for knockdowns can be found in Table S2. In drug treatment experiments, cells were plated for 24 hours prior to exposure to drug. AZ-628 was obtained from AstraZeneca. CI-1040 was obtained from Pfizer. R406 was synthesized in the Gray laboratory. BAY61-3606 and IKK VII were purchased from EMD Biosciences. BAY derivatives were synthesized for this study.

### Cell cycle analysis and cell viability assays

Cell cycle analysis was performed via FACS-based propidium iodide quantification, using standard methods. To measure cell viability, cells were grown in 96-well plates in the presence or absence of drug for 72 hours, fixed with 4% paraformaldehyde, and then stained with Syto60 (Invitrogen). Plates were imaged and quantified using the LiCor Odyssey system (LiCor).

### Bio-Plex signaling assays

The Bio-Plex assay system was used to measure signaling in drug-treated cells. Briefly, cells were incubated in the presence of drug for various amounts of time and then lysed in Bio-Rad cell lysis buffer (Bio-Rad). Protein quantification was performed using BCA assay (Pierce) and 5 µg of protein from each sample was used for Bio-Plex analysis. Phospho-signaling assays were performed using available phospho-signaling assay kits and quantified on a Bio-Plex 200 system (Bio-Rad): p-Iκβα (Ser32/Ser36), p-JNK (Thr183/Tyr185), p-MEK1 (Ser217/Ser221), p-ERK1/2 (Thr202/Tyr204, Thr185/Tyr187), p-p90RSK (Thr359/Ser363), p-p38 (Thr180/Tyr182), p-c-JUN (Ser63), p-ATF2 (Thr71), p-AKT (Ser473), p-S6 (Ser235/Ser236), p-STAT3 (Ser727), p-STAT3 (Tyr705), and p-GSK3α/β (Ser21/Ser9). Bio-Plex assay for total MEK1 was also performed as a loading control. All signals were normalized to a common control cell line lysate in order for assays between plates to be comparable.

### Biochemical activity assays

The biochemical activity of BAY61-3606 and derivatives were measured in two ways. First, we used Ambit's KINOMEscan™ technology to identify those kinases that are inhibited for substrate binding by the compounds, all assayed at 1 μM. Second, we used Invitrogen's SelectScreen® Biochemical Kinase Profiling Service to determine the *in vitro* IC50s for the compounds against specific kinases.

### Chemical derivation of BAY61-3603

Details on the synthesis of BAY derivatives, and the structures of those derivatives, can be found in Figure S5.

## Results

### AZ-628 and BAY61-3606 suppress growth in cells expressing K-RAS^G13D^

**Figure 1:**
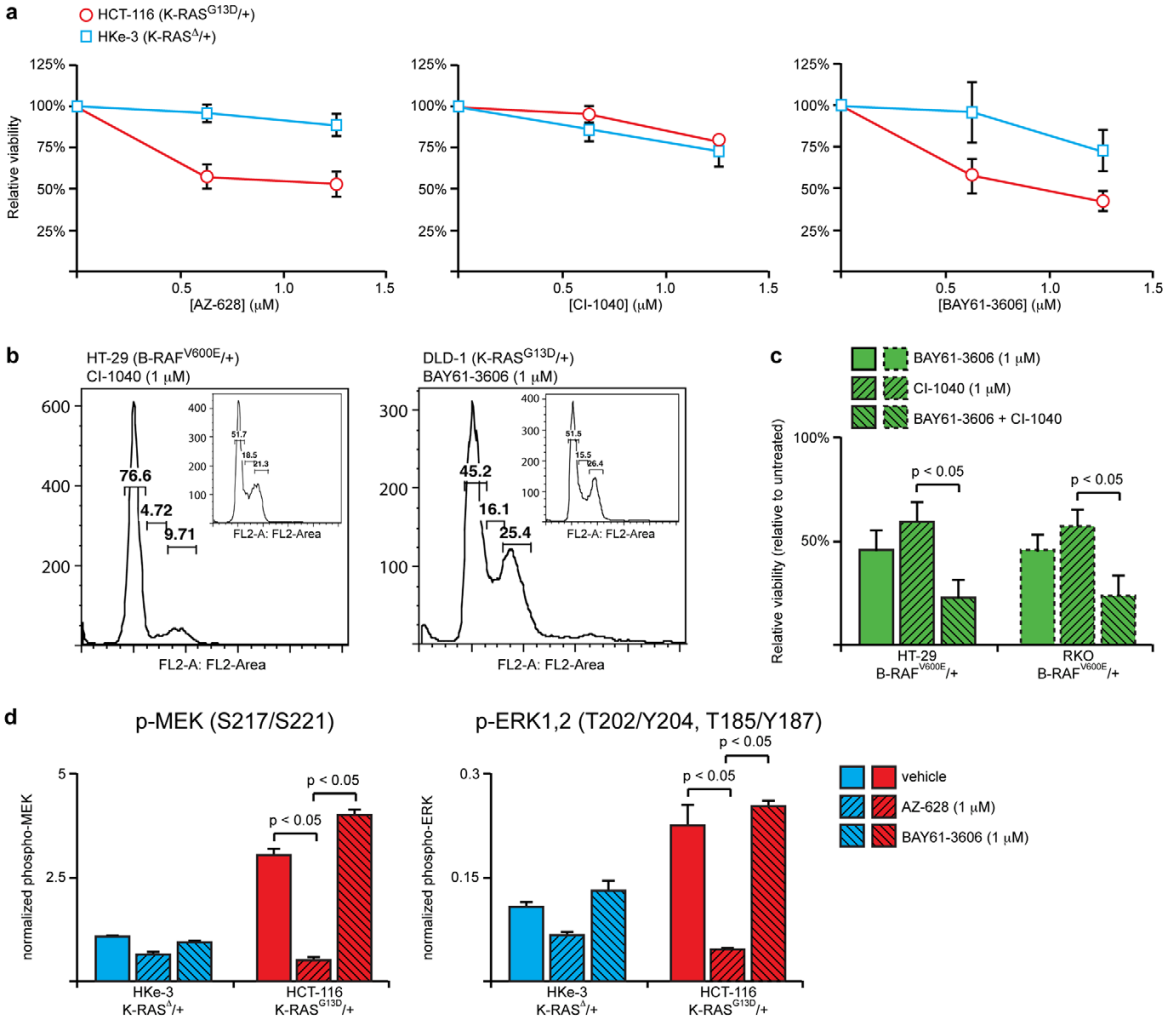
BAY61-3606 affects viability in cells expressing mutant K-RAS or B-RAF through a MAPK-independent pathway. (**a**) Cell viability quantified by Syto60 after 72 hours of AZ-628, CI-1040 or BAY61-3606 treatment in HCT-116 (K-RAS^G13D/+^, red) or HKe-3 (K-RAS^−/+^, blue) cell lines. Relative cell viability was normalized to DMSO vehicle treated control for each cell line. Error bars represent SEM for 3 independent experiments. Cells expressing mutant K-RAS were relatively sensitive to AZ-628 and BAY61-3606, but not CI-1040. (**b**) Cell cycle profiles, as determined by propidium iodide staining, of colorectal cancer cells with mutant B-RAF (HT-29) treated with CI-1040 or mutant K-RAS (DLD-1) treated with BAY61-3606. While inhibition of MEK induces G1 arrest in HT-29 cells, as evidenced by the loss of the 4N peak, BAY61-3606 did not appear to alter the profile of DLD-1 cells. (**c**) Cell viability of colorectal cancer cells expressing mutant B-RAF (V600E) after 72 hours of treatment with BAY61-3606 and/or CI-1040. Cells expressing mutant B-RAF (HT-29 – solid outline and RKO – dotted outline) were sensitive to both BAY61-3606 and CI-1040 and these two inhibitors cooperated to produce an enhanced response. (**d**) Phospho-MEK1 (Ser217/Ser221) and phospho-ERK1/2 (Thr202/Tyr204, Thr185/Tyr187) levels in K-RAS wild-type and mutant cells after 45 minute exposure to 1 µM AZ-628, 1 µM BAY61-3606, or vehicle control. Signals were measured using Bio-Plex assays. Relative signal was normalized to a master control lysate. AZ-628 treatment reduced the level of phospho-MEK and phospho-ERK, but BAY61-3606 did not.

In an effort to identify novel therapeutic targets for colorectal cancers expressing mutant K-RAS, we performed a screen for small molecule kinase inhibitors that affect viability in a genotype-specific manner. In these studies, we utilized a set of isogenic colon cancer cell lines that differ only in their K-RAS mutation status. The parental cell lines, HCT-116 and DLD-1, carry a heterozygous activating mutation in K-RAS (G13D/+). The derivative cell lines, HKe-3 and DKs-8, retain the wild-type allele, but have lost the mutant allele of K-RAS by virtue of gene targeting [17]. Consistent with our previous work [9], we found that K-RAS mutant cells were hypersensitive to AZ-628, a pan-RAF inhibitor [18], [19], compared to wild-type cells, but insensitive to CI-1040, a MEK inhibitor [6] (Fig. 1a). We also found that BAY61-3606, a Spleen Tyrosine Kinase (SYK) inhibitor, affected overall viability in HCT-116 and DLD-1 cells compared to HKe-3 and DKs-8 cells (Fig. 1a, Fig. S1a).

To determine how BAY61-3606 affected viability of cells expressing mutant K-RAS, we analyzed the cell cycle of cells treated with the drug. We found that BAY61-3606 did not alter the cell cycle profile of DLD-1 cells, nor did it induce apoptosis (Fig. 1b). In contrast to colorectal cancer cells expressing mutant K-RAS, cell lines that express mutant B-RAF were sensitive to inhibition of MEK and treatment with CI-1040 induced G1 arrest [6] (Fig. 1b, c). Interestingly, we found that B-RAF mutant cells lines were also sensitive to BAY61-3606 and that CI-1040 and BAY61-3606 cooperated to produce an enhanced negative effect on viability of cells expressing activated B-RAF (Fig. 1c). Taken together, these observations suggested (1) that BAY61-3606 inhibits a protein that is required for overall viability in cells expressing mutant K-RAS or B-RAF and (2) that BAY61-3606 targets a pathway that is independent of canonical MAPK/ERK signaling.

To test the second part of this hypothesis directly, we measured the activation state of MEK and ERK in cells that were treated with BAY61-3606. We found that inhibition of RAF with AZ-628 suppressed MEK and ERK phosphorylation, but that BAY61-3606 was unable to do so (Fig. 1d). Thus, while AZ-628 and BAY61-3606 both selectively affect viability in K-RAS mutant cells, they appear to do so through distinct pathways, with BAY61-3606 targeting a pathway that is independent of MEK and ERK.

### SYK is not the target of BAY61-3606 in cells expressing mutant K-RAS

**Figure 2:**
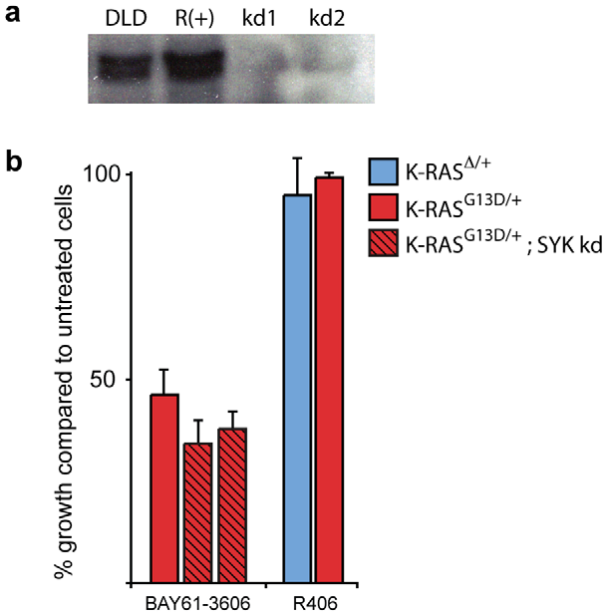
Inhibition of SYK is not responsible for the BAY61-3606 effect on cell viability in colorectal cancer cells. (**a**) Knockdown of SYK protein in DLD-1 cells via shRNA, as shown by Western Blotting. The Ramos (R) cell line, a hematopoetic cell line with a high expression of SYK, was used as a positive control. (**b**) Cell viability of DLD-1 cells (red), and its K-RAS wild-type (DKs-8 – blue) and SYK knockdown (red shaded) derivatives after 72 hours of treatment with 1 **μ**M of BAY61-3606 or R406, a distinct SYK inhibitor. Relative cell viability was normalized to DMSO vehicle treated control for each cell line. Error bars represent SEM for 3 independent experiments. DLD-1 cells and its K-RAS wild-type derivative did not exhibit sensitivity to R406, while knocking down SYK in DLD-1 cells only minimally affected its sensitivity to BAY61-3606.

Given that BAY61-3606 was originally developed as an ATP-competitive inhibitor of SYK [14], it was not surprising that it appeared to target a MEK/ERK-independent pathway. Nevertheless, HCT-116 cells, which are sensitive to BAY61-3606, do not express detectable levels of SYK, suggesting that it may not be the target of BAY61-3606 in this context. To explore this further, we used lentiviral shRNA to knock down SYK in DLD-1 cells (Fig. 2a). Knockdown of SYK did not affect overall growth rate nor did it significantly affect the response of DLD-1 cells to BAY61-3606 (Fig. 2b). Moreover, treatment of cells with R406, a structurally distinct inhibitor of SYK, did not result in a preferential effect in cells expressing mutant K-RAS (Fig. 2b). Taken together, these results suggested that SYK was not the relevant target of BAY61-3606 in colorectal cancer cells expressing mutant K-RAS.

### BAY61-3606 targets a small number of kinases

**Figure 3:**
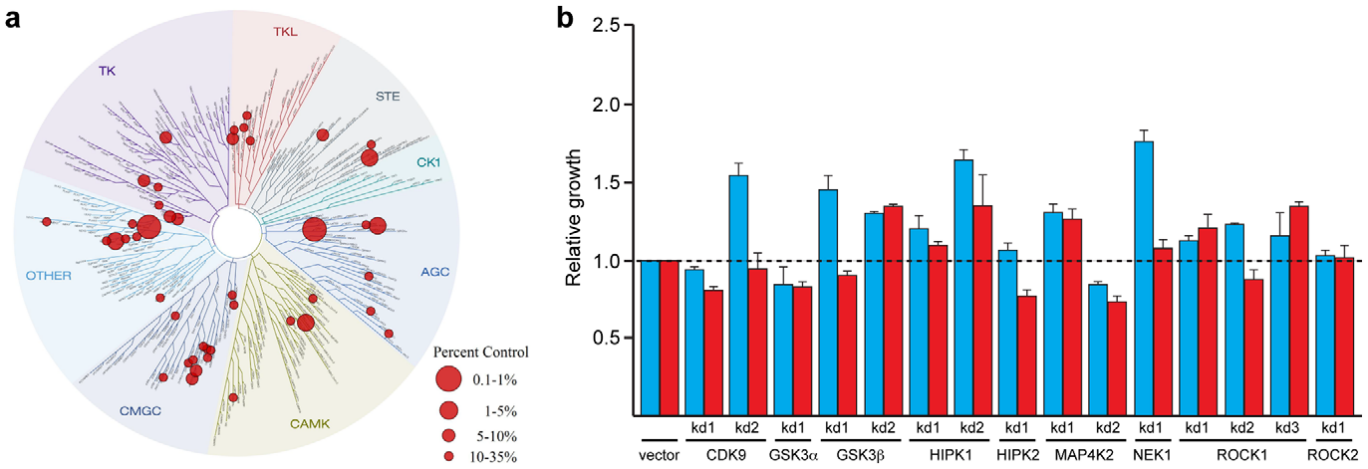
Identification and attempted validation of BAY61-3606 targets. (**a**) TREEspot image representing the inhibitory activity of BAY61-3606. Kinases that are inhibited for ATP binding by BAY61-3606 are indicated by red dots on the phylogenetic tree of kinases. The size of the red dot corresponds to the amount of inhibition by 1 μM BAY61-3606. The identity of the individual kinases can be found in Table S1. (**b**) Cell viability quantified by Syto60 after shRNA-mediated knockdown of the potential BAY61-3606 targets in DLD-1 (red) or DKs-8 (blue) cell lines. Relative cell viability is normalized to the parental cell lines infected with vector only. Error bars represent SEM for 2 independent experiments.

We hypothesized that the activity of BAY61-3606 in colon cancer cells was due to an “off-target” effect of the inhibitor, yet little was known about the promiscuity of this particular compound. To identify other potential targets of BAY61-3606, we first assayed the ability of BAY61-3606 to competitively inhibit active site binding in a panel of 402 kinases. We found that 1 μM BAY61-3606 inhibited binding by greater than 90% for only 15 kinases (Fig. 3a, Table 1, Table S1), indicating that it is a highly selective inhibitor, similar in selectivity to two clinical kinase inhibitors, Imatinib and Gefitinib [20]. Since inhibition of active site binding may, or may not, be directly correlated with inhibition of kinase activity, we performed a secondary analysis in which we determined the *in vitro* IC50 values for BAY61-3606 against many of these candidate targets. This study revealed MAP4K2, a STE-20 family kinase, as the kinase most sensitive to BAY61-3606, with an *in vitro* IC50 of 11.3 nM (Table 1).

**Table 1:** Activity of BAY61-3606 against selected kinases.

*Kinase*	*% inhibition ATP binding* *	*IC50 (nM)* **
CDK9	44	34.8
GSK3α	83	89.2
GSK3β	61	123
HIPK1	91.2	4240
HIPK2	91	248
MAP4K2	94	11.3
NEK1	73	159
ROCK1	96.8	144
ROCK2	87	166
SYK	93.9	25.5

* % inhibition of ATP binding as assessed by KINOMEscan™ (Ambit Biosciences).

** *In vitro* IC50 were determined by SelectScreen® (Invitrogen).

### Genetic analysis of potential BAY61-3606 targets

To follow-up on our biochemical analysis of BAY61-3606 activity, we used lentiviral shRNA to determine whether knockdown of any of the potential targets was able to phenocopy treatment with the drug (i.e. to selectively affect viability of K-RAS mutant cells) (Fig. S1c, Table S2). For these studies, we utilized the DLD-1/DKs-8 isogenic cell lines because we had used this pair for genetic analysis of SYK (Fig. 2). Knockdown of only one of the potential targets that we assayed, HIPK2, was able to selectively affect viability of K-RAS mutant cells (Fig. 3b). Nevertheless, we could only identify a single shRNA sequence that produced adequate knockdown of this target. As a result, this study was suggestive, but not conclusive, of a role for HIPK2 as a target of BAY61-3606 in cells expressing mutant K-RAS. We sought an independent way to identify the target of BAY61-3606.

### Identification of biologically active BAY61-3606 derivatives

**Figure 4:**
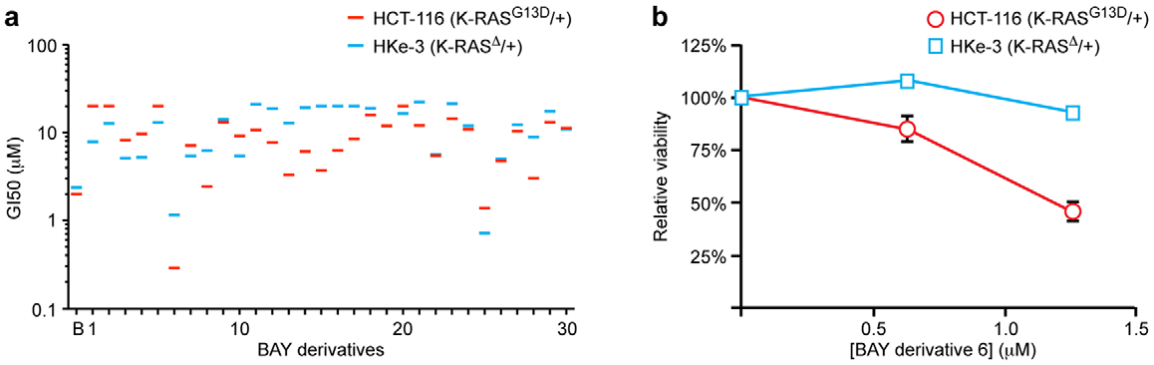
Biological activities of BAY61-3606 derivatives. (**a**) GI50 values for BAY61-3606 derivatives performed in HCT-116 (red) or HKe-3 (blue) cells. Derivative 6 was chosen for further study for its increased potency and specificity for K-RAS mutant cells. (**b**) Cell viability quantified by Syto60 after 72 hours exposure to BAY derivative 6 in HCT-116 (red) or HKe-3 (blue) cells. Relative cell viability was normalized to DMSO vehicle treated control for each cell line. Error bars represent SEM for 3 independent experiments. HCT-116 cells were relatively sensitive to BAY derivative 6.

Secondary to our genetic analysis, we took a chemical approach to studying BAY61-3606. In this study, we generated 30 structurally related derivatives of BAY61-3606 and assayed their ability to selectively affect viability of cells expressing mutant K-RAS (Fig. S2). Of these 30 derivatives, only one (derivative 6) retained its selectivity with potency similar to the parent compound (Fig. 4a, b, Fig. S1b). Others (e.g. derivative 8) retained selectivity but lost potency. To address the selectivity of BAY derivative 6 more broadly, we assessed its activity in a panel of colorectal cancer cell lines that were either mutant for wild-type for K-RAS. Consistent with our analysis of isogenic pairs, 4/5 cell lines expressing mutationally activated K-RAS responded to BAY derivative 6, while 0/3 cell lines expressing wild-type K-RAS responded (Fig. S3).

To compare with BAY61-3606, we assayed the ability of several derivatives to competitively inhibit active site binding in a large panel of kinases. Remarkably, each of the derivatives that were tested essentially lost their ability to competitively inhibit active site binding of the 402 kinases that were analyzed, including HIPK2 (Fig. S4). Consistent with this observation, *in vitro* activity assays revealed a loss of kinase inhibition activity for the two derivatives tested (Fig. S5).

### BAY61-3606 targets MAP4K2 to affect the response of wild-type cells to AZ-628

**Figure 5:**
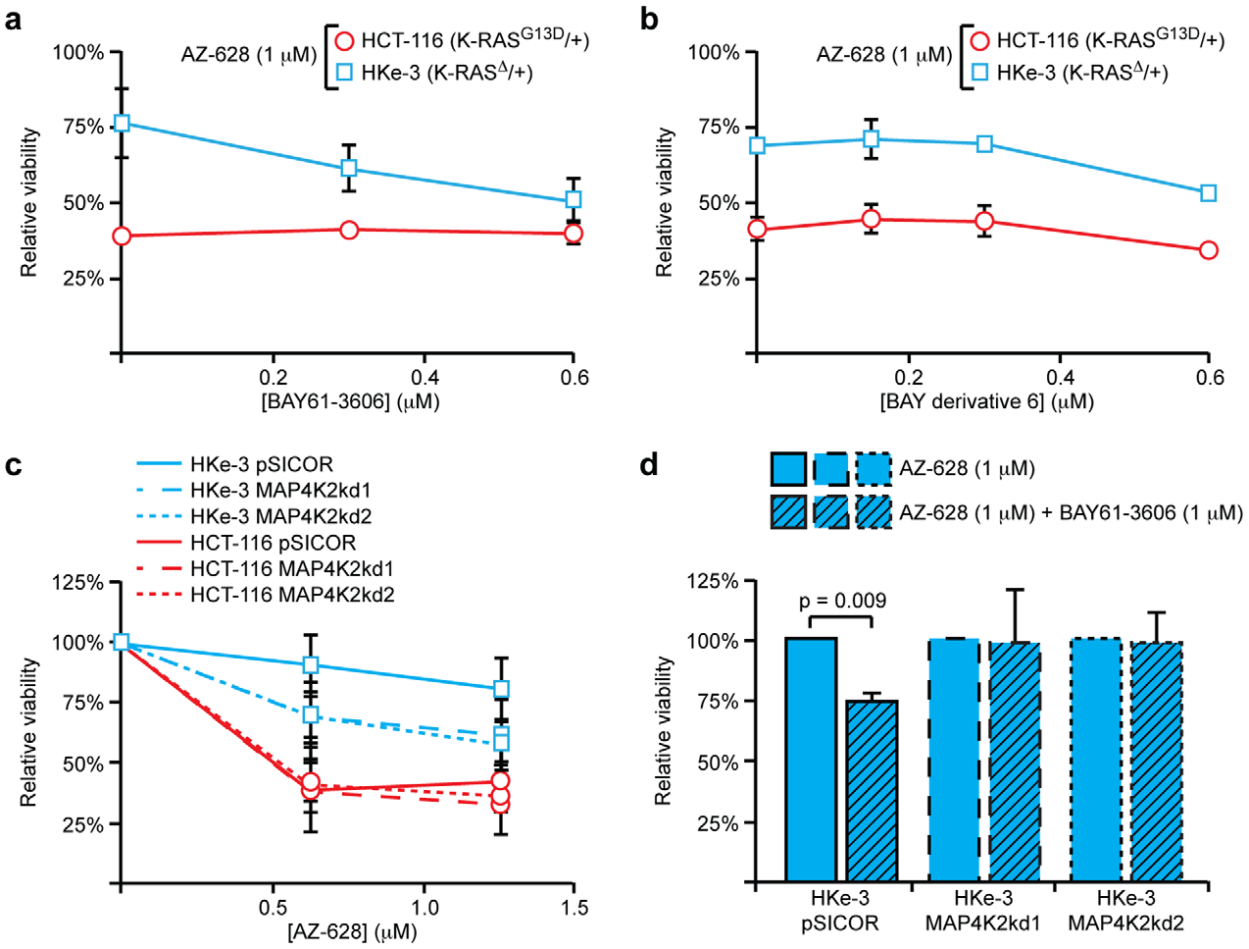
MAP4K2 is a target for BAY61-3606 that modulates the response of wild-type cells to AZ-628. (**a**) Cell viability quantified by Syto60 after 72 hours of combinatorial treatment with varying concentrations of BAY61-3606 and 1 µM AZ-628 in HCT-116 (red line) or HKe-3 (blue lines) cells. Relative cell viability was normalized to DMSO vehicle treated control for each cell line. Error bars represent SEM for 3 independent experiments. The two inhibitors cooperate in wild-type cells, but not in cells expressing mutant K-RAS. (**b**) Cell viability after 72 hours of combinatorial treatment with varying concentrations of BAY derivative 6 and 1 µM AZ-628 in HCT-116 and HKe-3 cells. BAY derivative 6 does not confer additional sensitivity to AZ-628 upon HKe-3 cells. (**c**) Cell viability quantified by Syto60 after 72 hours of AZ-628 treatment in HCT-116 or HKe-3 cell lines with MAP4K2 knockdown. Loss of MAP4K2 does not affect AZ-628 response in cells expressing mutant K-RAS, but enhances the effect of AZ-628 in cells expressing wild-type K-RAS. (**d**) Cell viability after 72 hours of combinatorial treatment with 1 µM BAY61-3606 and 1 µM AZ-628 (shaded) or 1 µM AZ-628 alone (clear) in HKe-3 cells with MAP4K2 knockdown. Relative cell viability was normalized to 1 µM AZ-628 treated samples. In parental HKe-3 cells, BAY61-3606 confers sensitivity to AZ-628. Upon loss of MAP4K2, BAY61-3606 no longer sensitizes.

In analyzing the relative activities of AZ-628 and BAY61-3606, we tested whether these two compounds would cooperate to produce an enhanced effect in cells expressing mutant K-RAS, similar to the cooperative effect seen with CI-1040 and BAY61-3606 in cells expressing mutant B-RAF. AZ-628 and BAY61-3606 did not cooperate in HCT-116 cells (Fig. 5a), however, suggesting that they may target a common pathway. Interestingly these two inhibitors did cooperate to negatively affect viability of cells expressing wild-type K-RAS. In essence, HKe-3 cells that were formally resistant to AZ-628 became sensitive when they were also treated with BAY61-3606 (Fig. 5a). BAY derivative 6, which retained its ability to selectively suppress proliferation in cells expressing mutant K-RAS (Fig. 4b), lost its ability to cooperate with AZ-628 in cells expressing wild-type K-RAS (Fig. 5b).

With the exception of SYK, BAY61-3606 was more than 10-fold more effective at inhibiting MAP4K2 than any other kinase. Moreover, BAY derivative 6 lost its ability to effectively inhibit the *in vitro* kinase activity of MAP4K2 (Fig. S5). Based on these observations, we explored whether MAP4K2 plays a role in the sensitivity of wild-type and K-RAS mutant cells to BAY61-3606. HKe-3 and Dks-8 cells lacking MAP4K2 were hypersensitive to AZ-628, while MAP4K2 knockout had no effect on the response of HCT-116 or DLD-1 cells (Fig. 5c, Fig. S6a). We reasoned that if inhibition of MAP4K2 by BAY61-3606 accounted for the altered response of wild-type cells to AZ-628, then K-RAS wild-type cells lacking MAP4K2 would not be further sensitized to AZ-628 by treatment with BAY61-3606. As predicted, BAY61-3606 and AZ-628 failed to cooperatively affect viability in HKe-3 cells lacking MAP4K2 (Fig. 5d). The results strongly suggest that BAY61-3606 alters the response of cell expressing wild-type K-RAS to AZ-628 by inhibiting the kinase activity of MAP4K2.

### MAP4K2 activates the NFκB pathway to counteract AZ628-dependent growth inhibition

**Figure 6:**
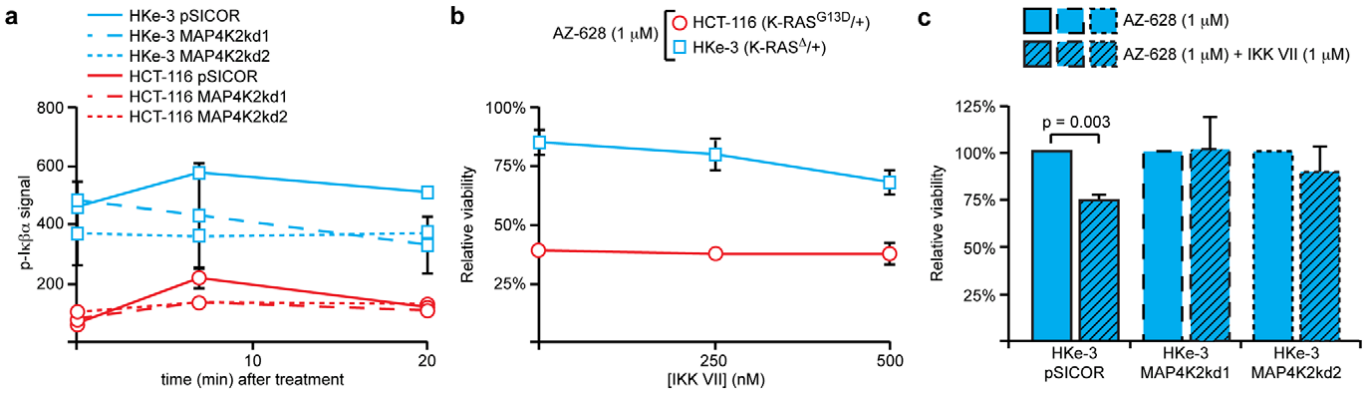
MAP4K2 modulates NFκβ signaling. (**a**) Time course of phospho-Iκβα (Ser32/Ser36) activation after 1 µM AZ-628 treatment in HCT-116 (red lines) or HKe-3 (blue line) cells with MAP4K2 knock down, as measured via Bio-Plex. Relative signal was normalized to a master control lysate. Error bars represent SEM for 3 independent experiments. NFκβ signaling was enhanced in HKe-3 cells after exposure to AZ-628 and this was dependent upon MAP4K2. (**b**) Cell viability quantified by Syto60 after 72 hours of combinatorial treatment with IKK inhibitor VII and 1 µM AZ-628. Relative cell viability was normalized to DMSO vehicle treated control for each cell line. Like BAY61-3606, IKK inhibitor VII enhanced the effect of AZ-628 specifically in K-RAS wild-type cells. (**c**) Cell viability after 72 hours of combinatorial treatment of 1 µM IKK inhibitor VII and 1 µM AZ-628 (shaded) or 1 µM AZ-628 alone (clear) in HKe3 cells with MAP4K2 knockdown. Relative cell viability was normalized to 1 µM AZ-628 treated samples. Loss of MAP4K2 abrogated the ability of IKK inhibitor VII to sensitize HKe-3 cells to AZ-628.

Given that MAP4K2 is important for the response of wild-type cells to AZ-628, we next asked how MAP4K2 functions to regulate the response to RAF inhibition. To identify the pathway responsible for MAP4K2 action, we used Bio-Plex phospho-protein analysis to measure the effects of MAP4K2 knockdown on various cellular signaling pathways. In total, we profiled 13 phospho-proteins: Iκβα (Ser32/Ser36), JNK (Thr183/Tyr185), MEK1 (Ser217/Ser221), ERK1/2 (Thr202/Tyr204, Thr185/Tyr187), RSK (Thr359/Ser363), p38 (Thr180/Tyr182), c-JUN (Ser63), ATF2 (Thr71), AKT (Ser473), S6 (Ser235/Ser236), STAT3 (Ser727), STAT3 (Tyr705), and GSK3α/β (Ser21/Ser9) (Fig. S7). We found two differences between HKe-3 and HCT-116 that we posited might be related to the function of MAP4K2. First, we found that the basal level of Iκβα phosphorylation was significantly lower in HCT-116 than in HKe-3 cells, suggesting that NFκβ signaling is suppressed in cells expressing activated K-RAS (Fig. S7). Iκβα phosphorylation was further induced in HKe-3 cells after treatment with AZ-628 and this induction was dependent upon MAP4K2 (Fig. 6a). This observation is consistent with previous studies linking MAP4K2 to NFκβ signaling [21]. Second, we found that JNK was strongly activated in HKe-3 cells after treatment with AZ-628 (Fig. S7). Although MAP4K2 has been previously linked to JNK signaling, this activation did not appear to require MAP4K2 (Fig. S6b).

Since NFκβ was appeared to hyper-activated in wild-type cells in a MAP4K2-dependent manner, we surmised that inhibition of the NFκβ pathway would have the same effect as BAY61-3606 or MAP4K2 knockdown. That is, we expected that inhibition of NFκβ would increase the sensitivity of K-RAS wild-type cells to AZ-628 without affecting the sensitivity of K-RAS mutant cells. As predicted, inhibition of NFκβ increased the sensitivity of HKe-3 and Dks-8 cells to AZ-628, essentially phenocopying MAP4K2 knockdown (Fig. 6b, Fig. S6c). By contrast, inhibition of JNK did not alter the sensitivity of HKe-3 cells to AZ-628 (Fig. S6d). Further, as with BAY61-3606 treatment, knockdown of MAP4K2 abolished the ability of NFκβ inhibition to sensitize wild-type cells to AZ-628 (Fig. 6c). From these data, we conclude that MAP4K2 functions upstream in the NFκβ pathway to regulate the response of colorectal cancer cells to inhibition of RAF.

## Discussion

K-RAS is mutationally activated in approximately 40% of colorectal cancers [2]. Activated K-RAS is thought to confer oncogenicity via its canonical downstream signaling pathways, for example the RAF-MEK-ERK (MAPK) signaling cascade. Consistent with this idea, activating mutations in B-RAF occur in 15% of colorectal cancers and they are mutually exclusive with K-RAS mutations [22]. Nevertheless, inhibition of MAPK signaling, typically by directly inhibiting MEK, has been largely ineffective in treating K-RAS mutant colorectal cancer [9], [23]. The lack of efficacy of MEK inhibitors in this context may be due to the pleiotropic function of K-RAS, which has been shown to promote transformation through the PI3K and RAL effector pathways in addition to MAPK [24], [25]. Yet, PI3K pathway mutations also occur in colorectal cancers and are often coincident with K-RAS mutations, suggesting that PI3K is not a common effector of K-RAS signaling in colon cancer [26]. And while PI3K mutations have been associated with resistance to MEK inhibitors in cancer cell lines [27], [28], K-RAS mutant colon cancers from genetically engineered mice are intrinsically resistant to inhibition of MEK [9]. Our data are consistent with an alternative explanation for the lack of efficacy of MEK inhibitors in colorectal cancers expressing mutant K-RAS – that there exists an alternate/parallel pathway downstream of B-RAF that mediates K-RAS-induced oncogenicity.

In our study, we characterized the activity of a small molecule, BAY61-3606, that preferentially affected viability in colorectal cancer cells expressing mutant K-RAS compared to isogenic cells expressing only wild-type K-RAS (Fig. 1a, Fig. S1a). Since BAY61-3606 is an ATP-competitive kinase inhibitor, its ability to preferentially affect cells expressing mutant K-RAS initially suggested that it targets a kinase functioning downstream of K-RAS to promote proliferation. We have previously shown that K-RAS promotes colon cancer cell proliferation through a RAF-dependent, but MEK-independent, signaling pathway [9]. Three pieces of evidence implicate BAY61-3606 as an inhibitor of this MEK-independent pathway downstream of RAF. First, BAY61-3606 did not cooperate with AZ-628 in cells expressing mutant K-RAS (Fig. 5a), suggesting that these inhibitors targeted a common pathway. Second, BAY61-3606 affected growth in KRAS mutant cells, but, unlike AZ-628, did not affect the phosphorylation state of MEK or ERK (Fig. 1d). Finally, BAY61-3606 slowed the growth of colorectal cancer cells expressing mutant B-RAF and cooperated with a MEK inhibitor to produce an enhanced response in these cells (Fig. 1c).

Although BAY61-3606 was initially characterized as an ATP competitive kinase inhibitor, its selectivity for decreasing the viability of K-RAS mutant cells may not require this activity. Our studies of BAY61-3606 derivatives demonstrate that those lacking the ability to affect active site binding can still maintain their ability to selectively affect cell viability. One possible explanation for this observation is that the relevant target of BAY61-3606 and its biologically active derivatives is a non-kinase protein. Aside from inhibiting kinases, ATP analogs can affect biology through other processes, including nucleic acids synthesis [29], [30] and microtubule motor transport [31]. Alternately, the relevant target may not be among the 402 kinases that were surveyed in our assay or BAY61-3606 and derivatives could inhibit kinase activity without affecting active site binding. If so, they would not score in our screen.

In addition to its activity in cells expressing mutant K-RAS or B-RAF, we also identified a secondary biological effect of BAY61-3606; it conferred upon wild-type cells, which are normally resistant to AZ-628, sensitivity to RAF inhibition (Fig. 5a). Using a variety of approaches, we identified MAP4K2 as the target for BAY61-3606 in wild-type cells. MAP4K2 (also known as GCK, for Germinal Center Kinase) is a member of the STE20 family of protein kinases [32]. MAP4K2 has been shown to play a role in the inflammatory process and is activated by inflammatory stimuli such as TNFα, IL-1, and LPS [33], [34]. MAP4K2 interacts with TNFR-associated factor 2 (TRAF2) and MAPK/ERK kinase kinase 1 (MEKK1), thereby linking TNF signaling to activation of the pro-death JNK and p38 MAPK pathways [35]. Aside from activating pro-death signaling pathways, the TNF receptor pathway can also promote cell survival through the NFκβ pathway. Incidentally, the NFκβ pathway is recruited by the TRAF2 complex upon TNFR activation [36]. Moreover, MAP4K2 has been shown to positively regulate NFκβ to protect melanoma cells from UV-induced apoptosis [21].

Inhibition of RAF by AZ-628 leads to MAP4K2-dependent activation of NFκβ signaling in wild-type cells, presumably to ensure cell survival (Fig. 6a). To maintain reliable behavior, cell signaling networks have evolved robust feedback mechanisms in order to minimize the effects of focal perturbations, which can arise from various stresses that cells encounter. The robustness of a signaling network is evident in its compensatory behavior given the activation or inactivation of a single pathway. For example, it has been demonstrated that RAF pathway activity is anti-correlated to PI3K signaling through global network feedback [37]. Consequently, pharmacologic inhibition of MEK results in PI3K pathway activation, resulting in cell survival [38]. Similarly, inhibition of mTOR, a downstream effector of PI3K, has been shown to increase ERK activity [39]. Given the large number of mutations that cancer cells accumulate, oncogenic signaling networks have evolved to be very different from signaling networks in normal cells. Whereas cancer cells have evolved such that they are robust to growth and proliferation, there may be areas in their networks that are more fragile to perturbations than their wild-type counterparts, which can be exploited for therapeutic purposes.

While our studies specifically address the function of the small molecule kinase inhibitor BAY61-3606 in colorectal cancer cells, they more generally address the varied activities that kinase inhibitors can have in cancer cells. Although inhibitor promiscuity is often viewed in a negative light, the ability of a given small molecule to target multiple kinases may, in fact, be beneficial for targeting diverse genotypic classes of cancer.

## Supporting Information

Figure S1 **Genetic analysis of BAY61-3606 response in DLD-1 cells.** (a) Cell viability quantified by Syto60 after 72 hours of BAY61-3606 treatment in DLD-1 (K-RAS^G13D/+^, red) or DKs-8 (K-RAS^−/+^, blue) cell lines. Relative cell viability was normalized to DMSO vehicle treated control for each cell line. Error bars represent SEM for 3 independent experiments. The differential response in the two cell lines is statistically significant (p = 0.019 at 1 μM). (b) Cell viability quantified by Syto60 after 72 hours of BAY derivative 6 treatment in DLD-1 (K-RAS^G13D/+^, red) or DKs-8 (K-RAS^−/+^, blue) cell lines. Relative cell viability was normalized to DMSO vehicle treated control for each cell line. Error bars represent SEM for 3 independent experiments. (c) Validation of shRNAs. Relative gene expression of shRNA-mediated knockdowns of potential BAY61-3606 targets in DLD-1 (red) and DKs-8 (blue) cells. Gene expression is measured via Taqman assay and calculated using standard methods in reference to the housekeeping gene TBP. Error bars represent SEM for 3 independent experiments.
(PDF)

Figure S2 **Chemical derivation of BAY61-3606 derivatives.** (a) Synthesis of BAY derivative 6. To a stirred solution of 5,7-dichloroimidazo [1,5-*f*] pyrimidine (186.0 mg, 1.0 mmol) in DMF (5.0 mL) was added 2-methoxybenzenamine (123.0 mg, 1.0 equiv). After 1 h heating at 70°, the mixture was purified on silica gel column with methylene chloride and methanol (10∶1) as eluent to give of 7-chloro-N-(2-methoxyphenyl)imidazo [1,5-*f*] pyrimidin-5-amine(245 mg, yield 89%). To a solution of 7-chloro-N-(2-methoxyphenyl)imidazo [1,5-*f*] pyrimidin-5-amine (200.0 mg, 0.73 mmol) and 3,4-dimethoxyphenylboronic acid (160.0 mg, 1.2 equiv) in 5.0 mL 1,4-dioxane was added Bis(triphenylphosphine) palladium(II) dichloride (51.0 mg, 0.1 equiv) as catalyst and saturated potassium carbonate aqueous solution (2.0 mL) as base. The mixture was heated for 2 h at 80° and then was diluted with chloroform and 2-propanol (50 mL, 4∶1). The organic layer was washed with water, brine and was dried with sodium sulfate. After removal of solvent, the crude was purified by column with methylene chloride and methanol (10∶1) to give BAY derivative 6 (192.0 mg, 70%). ^1^H NMR (DMSO-d_6_) 9.05 (s, 1 H), 8.22 (s, 1 H), 7.23 (d, *J* = 7.8 Hz, 1 H), 7,61-7.53 (m, 4 H), 7.26 (t, *J* = 7.8 Hz, 1 H), 7.16 (d, *J* = 8.4 Hz, 1 H), 7.04 (t, *J* = 7.8 Hz, 1 H), 6.94 (d, *J* = 8.4 Hz, 1 H), 3.80 (s, 3 H), 3.75 (s, 3 H), 3.72 (s, 3 H). (b) Chemical structures of all BAY derivatives.
(PDF)

Figure S3 **Evaluation of BAY derivative 6 activity in colorectal cancer cell lines.** Cell viability quantified by Syto60 after 72 hours of BAY derivative 6 treatment in 5 cell lines expressing mutant K-RAS and in 3 cell lines expressing wild-type K-RAS. Relative cell viability was normalized to an untreated control for each cell line. Error bars represent SEM for 3 independent experiments. With the exception of GP5d (highlight in bold red), all of the cell lines expressing mutant K-RAS respond to BAY derivative 6.
(PDF)

Figure S4 **BAY61-3606 derivatives lose ATP competitive activity.** TREEspot images for five different derivatives of BAY61-3606. Both inhibitors that retained selectivity for K-RAS mutant cells (e.g. 6 and 8) and those that lost selectivity (e.g. 1, 21, and 28), failed to effectively inhibit ATP binding by the majority of kinases that were assayed.
(PDF)

Figure S5 **Kinase inhibition profiles of BAY61-3606 and its derivatives.** Inhibitor activity was measured using Invitrogen's SelectScreen® Biochemical Kinase Profiling Service.
(PDF)

Figure S6 **Evaluation of MAP4K2 in the BAY61-3606 response.** (a) Cell viability quantified by Syto60 after 72 hours of AZ-628 treatment in DLD-1 or DKs-8 cell lines with MAP4K2 knockdown. Loss of MAP4K2 does not affect AZ-628 response in cells expressing mutant K-RAS, but enhances the effect of AZ-628 in cells expressing wild-type K-RAS. (b) Time course of phospho-JNK (Thr183/Tyr185) after 1 µM AZ-628 treatment in HCT-116 (red lines) or HKe-3 (blue line) cells with MAP4K2 knock down, as measured by Bio-Plex. Relative signal was normalized to a master control lysate. Error bars represent SEM for 3 independent experiments. JNK signaling was enhanced in HKe-3 cells but was independent of MAP4K2. (c) Cell viability quantified by Syto60 after 72 hours of combinatorial treatment with IKK inhibitor VII and 1 µM AZ-628. Relative cell viability was normalized to DMSO vehicle treated control for each cell line. Like BAY61-3606, IKK inhibitor VII enhanced the effect of AZ-628 specifically in K-RAS wild-type cells. (d) Cell viability quantified by Syto60 after 72 hours of combinatorial treatment with the JNK inhibitor SP600125 and 1 µM AZ-628. Relative cell viability was normalized to DMSO vehicle treated control for each cell line. Unlike BAY61-3606, SP600125 did not affect AZ-628 sensitivity in K-RAS wild-type cells.
(PDF)

Figure S7 **Signaling pathway activity in response to AZ-628.** Phospho-protein measurements were made after 45 minutes of exposure to 1 μM AZ-628 or DMSO vehicle control treatment in HCT-116 (red squares) or HKe-3 (blue dots) cells. X's represent measurements from cells with MAP4K2 knock down. All measurements were quantified by Bio-Plex signaling assays. Relative signal was normalized to a master control lysate.
(PDF)

Table S1 **Active site binding inhibition data.**
(PDF)

Table S2 **shRNAs used in this study.**
(PDF)
