# Supplementary figures and images for: BAY61-3606 Affects the Viability of Colon Cancer Cells in a Genotype-Directed Manner
Source: PLoS One. 2012 Jul 18;7(7):e41343. doi: 10.1371/journal.pone.0041343 (PMC3399817; doi:10.1371/journal.pone.0041343)

Figure S1

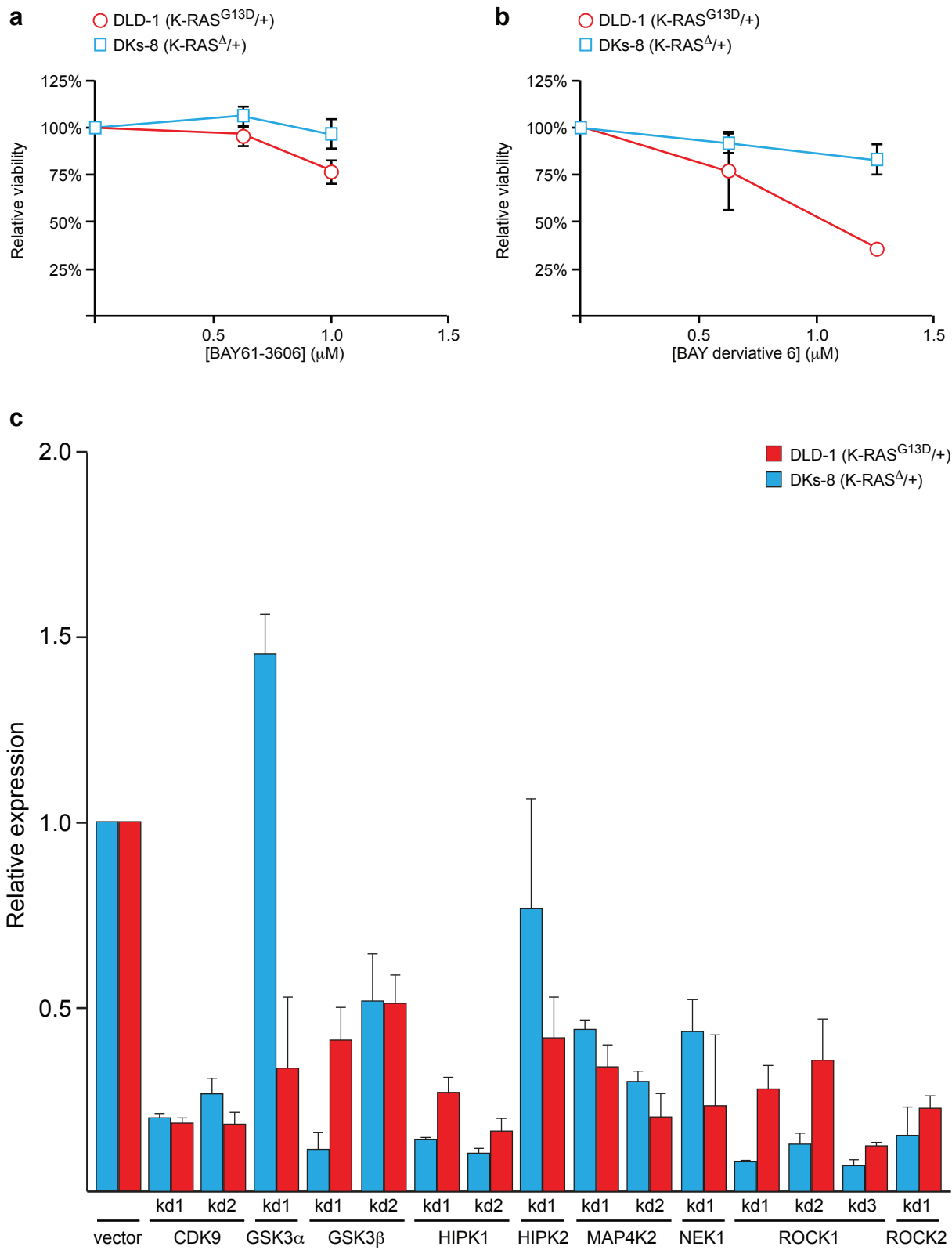

Supplement: Figure S1 — Genetic analysis of BAY61-3606 response in DLD-1 cells. (a) Cell viability quantified by Syto60 after 72 hours of BAY61-3606 treatment in DLD-1 (K-RASG13D/+, red) or DKs-8 (K-RAS−/+, blue) cell lines. Relative cell viability was normalized to DMSO vehicle treated control for each cell line. Error bars represent SEM for 3 independent experiments. The differential response in the two cell lines is statistically significant (p = 0.019 at 1 μM). (b) Cell viability quantified by Syto60 after 72 hours of BAY derivative 6 treatment in DLD-1 (K-RASG13D/+, red) or DKs-8 (K-RAS−/+, blue) cell lines. Relative cell viability was normalized to DMSO vehicle treated control for each cell line. Error bars represent SEM for 3 independent experiments. (c) Validation of shRNAs. Relative gene expression of shRNA-mediated knockdowns of potential BAY61-3606 targets in DLD-1 (red) and DKs-8 (blue) cells. Gene expression is measured via Taqman assay and calculated using standard methods in reference to the housekeeping gene TBP. Error bars represent SEM for 3 independent experiments. (PDF) [file pone.0041343.s001.pdf]

Figure S2

**a**

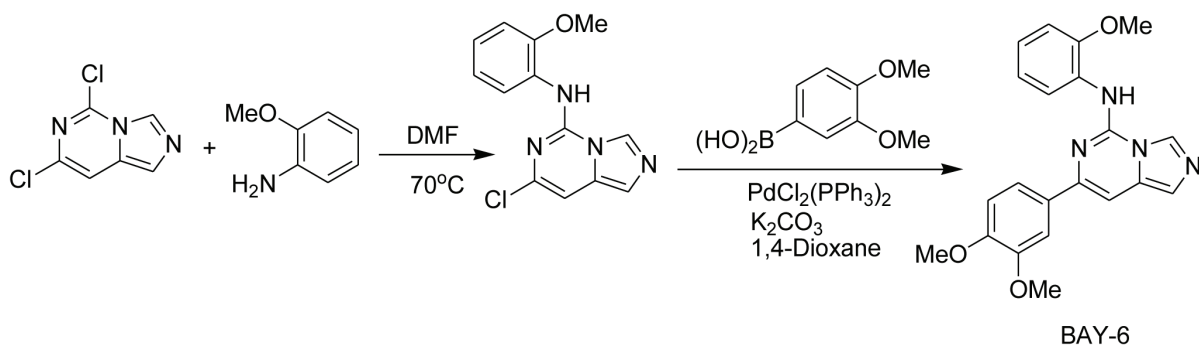

**b**

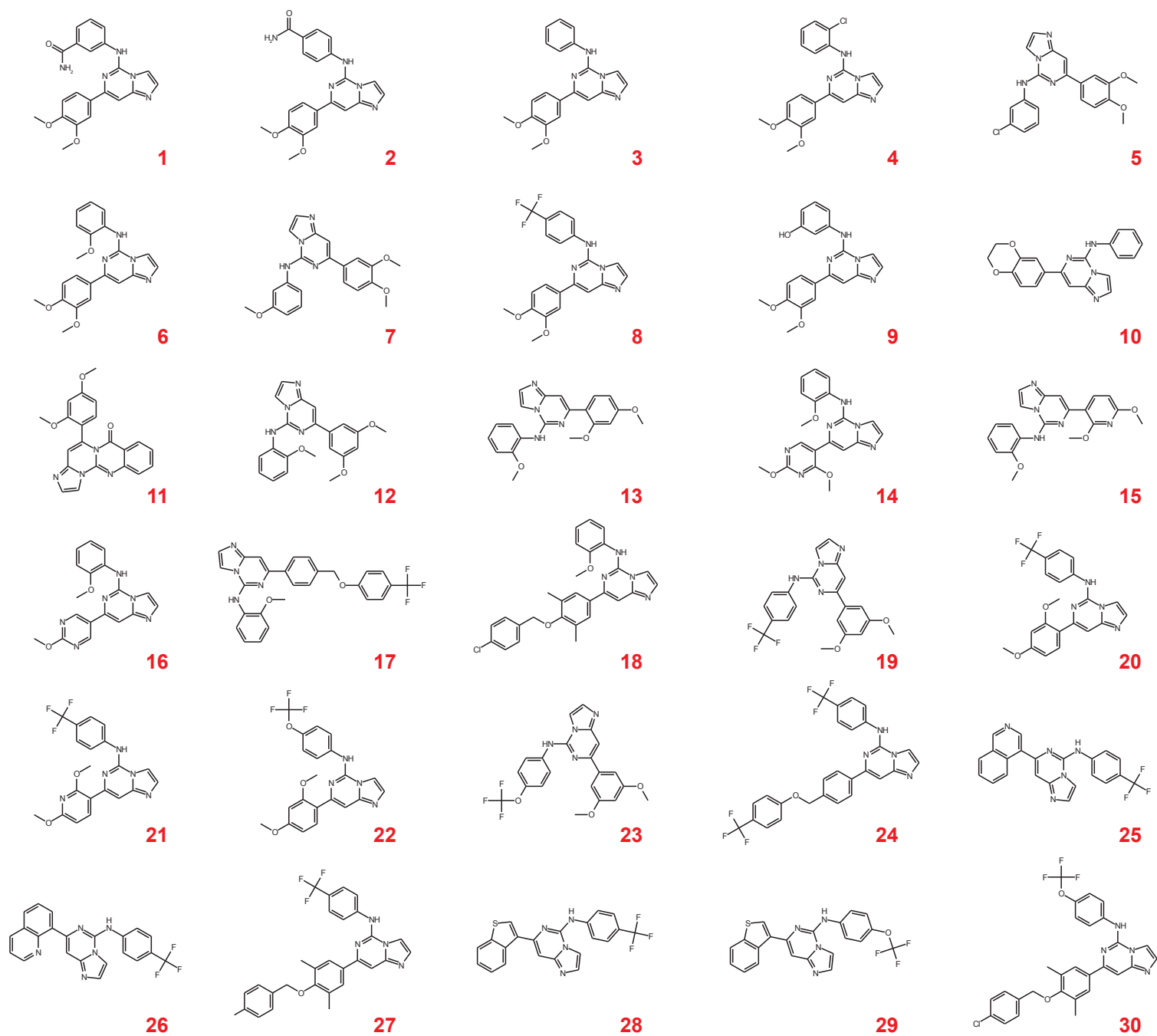

Supplement: Figure S2 — Chemical derivation of BAY61-3606 derivatives. (a) Synthesis of BAY derivative 6. To a stirred solution of 5,7-dichloroimidazo [1,5-f] pyrimidine (186.0 mg, 1.0 mmol) in DMF (5.0 mL) was added 2-methoxybenzenamine (123.0 mg, 1.0 equiv). After 1 h heating at 70°, the mixture was purified on silica gel column with methylene chloride and methanol (10∶1) as eluent to give of 7-chloro-N-(2-methoxyphenyl)imidazo [1,5-f] pyrimidin-5-amine(245 mg, yield 89%). To a solution of 7-chloro-N-(2-methoxyphenyl)imidazo [1,5-f] pyrimidin-5-amine (200.0 mg, 0.73 mmol) and 3,4-dimethoxyphenylboronic acid (160.0 mg, 1.2 equiv) in 5.0 mL 1,4-dioxane was added Bis(triphenylphosphine) palladium(II) dichloride (51.0 mg, 0.1 equiv) as catalyst and saturated potassium carbonate aqueous solution (2.0 mL) as base. The mixture was heated for 2 h at 80° and then was diluted with chloroform and 2-propanol (50 mL, 4∶1). The organic layer was washed with water, brine and was dried with sodium sulfate. After removal of solvent, the crude was purified by column with methylene chloride and methanol (10∶1) to give BAY derivative 6 (192.0 mg, 70%). 1H NMR (DMSO-d6) 9.05 (s, 1 H), 8.22 (s, 1 H), 7.23 (d, J = 7.8 Hz, 1 H), 7,61-7.53 (m, 4 H), 7.26 (t, J = 7.8 Hz, 1 H), 7.16 (d, J = 8.4 Hz, 1 H), 7.04 (t, J = 7.8 Hz, 1 H), 6.94 (d, J = 8.4 Hz, 1 H), 3.80 (s, 3 H), 3.75 (s, 3 H), 3.72 (s, 3 H). (b) Chemical structures of all BAY derivatives. (PDF) [file pone.0041343.s002.pdf]

Figure S3

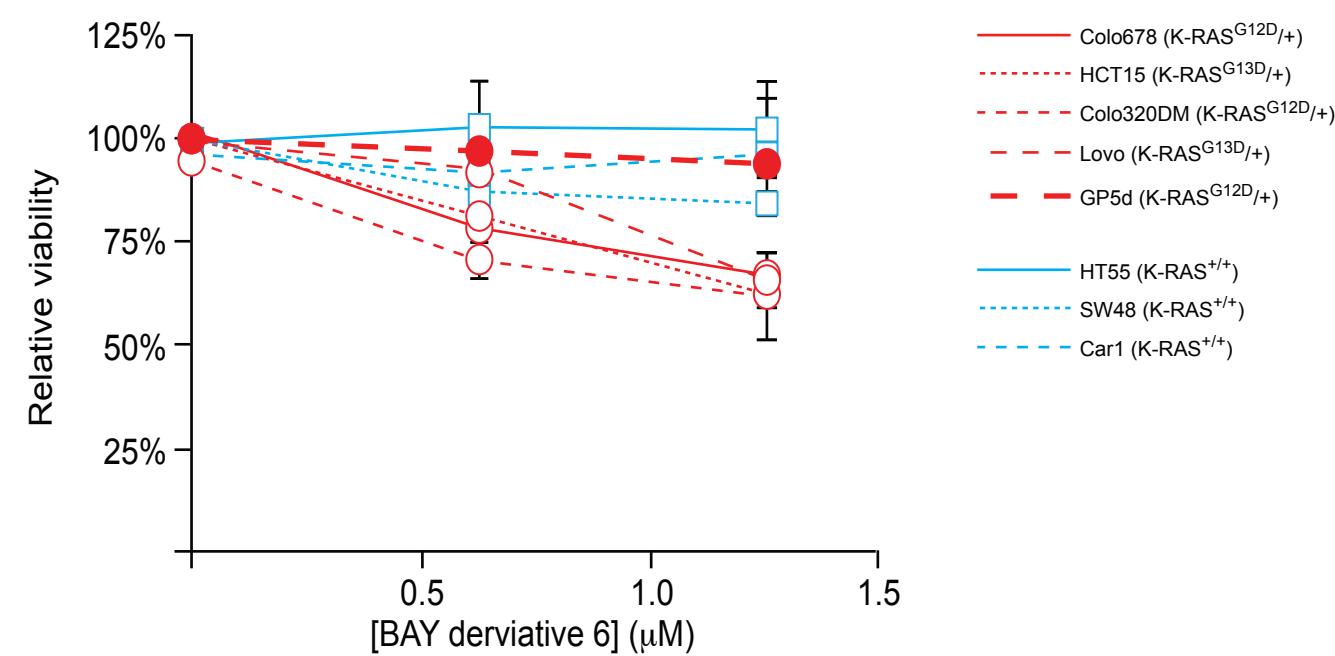

Supplement: Figure S3 — Evaluation of BAY derivative 6 activity in colorectal cancer cell lines. Cell viability quantified by Syto60 after 72 hours of BAY derivative 6 treatment in 5 cell lines expressing mutant K-RAS and in 3 cell lines expressing wild-type K-RAS. Relative cell viability was normalized to an untreated control for each cell line. Error bars represent SEM for 3 independent experiments. With the exception of GP5d (highlight in bold red), all of the cell lines expressing mutant K-RAS respond to BAY derivative 6. (PDF) [file pone.0041343.s003.pdf]

Figure S4

derivative 1

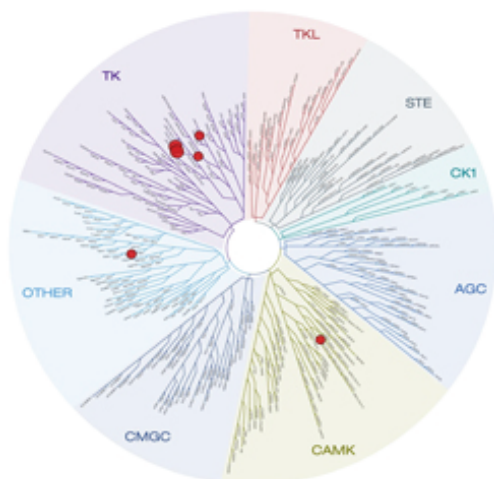

derivative 6

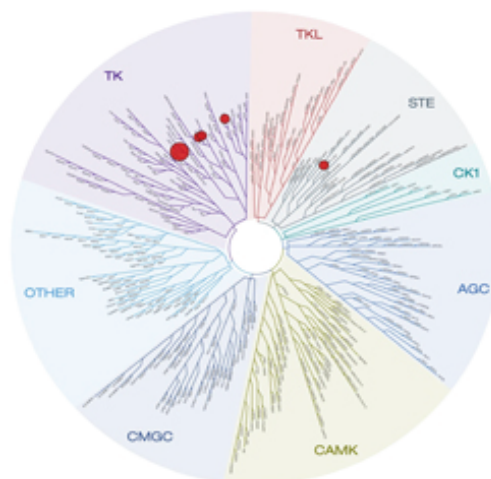

derivative 8

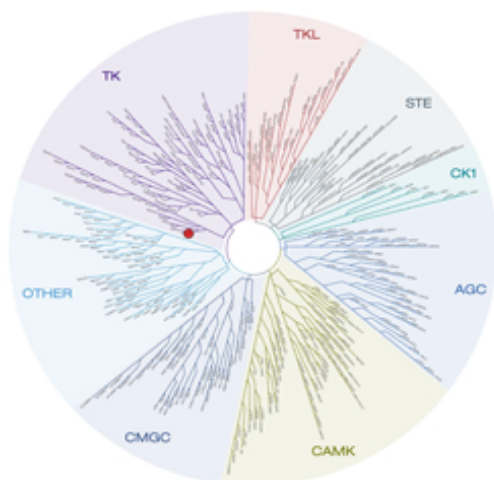

derivative 21

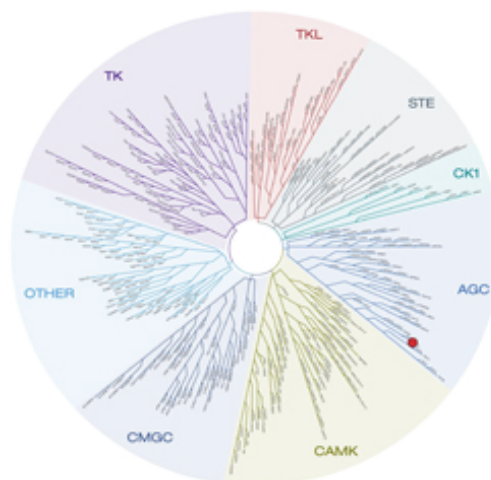

derivative 28

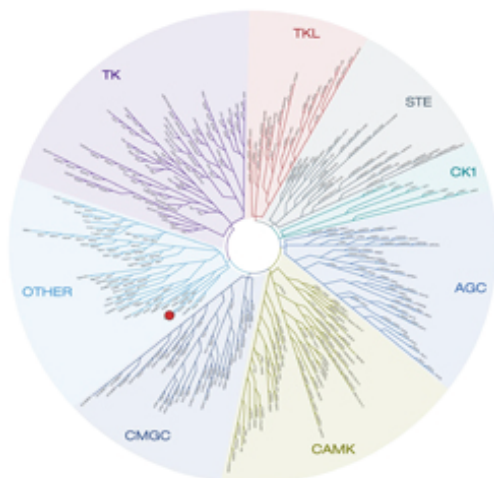

Percent Control

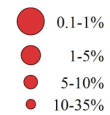

Supplement: Figure S4 — BAY61-3606 derivatives lose ATP competitive activity. TREEspot images for five different derivatives of BAY61-3606. Both inhibitors that retained selectivity for K-RAS mutant cells (e.g. 6 and 8) and those that lost selectivity (e.g. 1, 21, and 28), failed to effectively inhibit ATP binding by the majority of kinases that were assayed. (PDF) [file pone.0041343.s004.pdf]

Figure S5

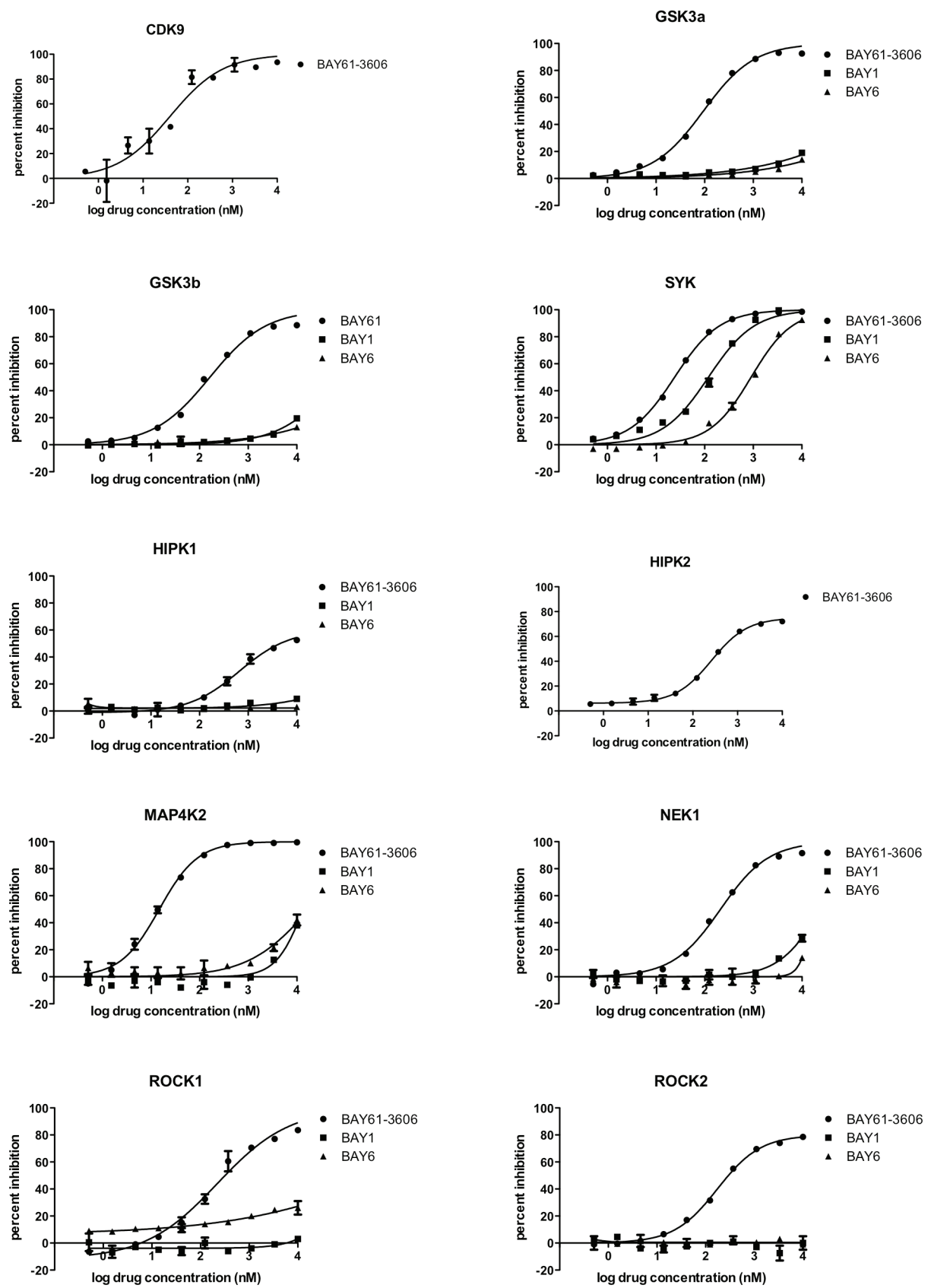

Supplement: Figure S5 — Kinase inhibition profiles of BAY61-3606 and its derivatives. Inhibitor activity was measured using Invitrogen's SelectScreen® Biochemical Kinase Profiling Service. (PDF) [file pone.0041343.s005.pdf]

Figure S6

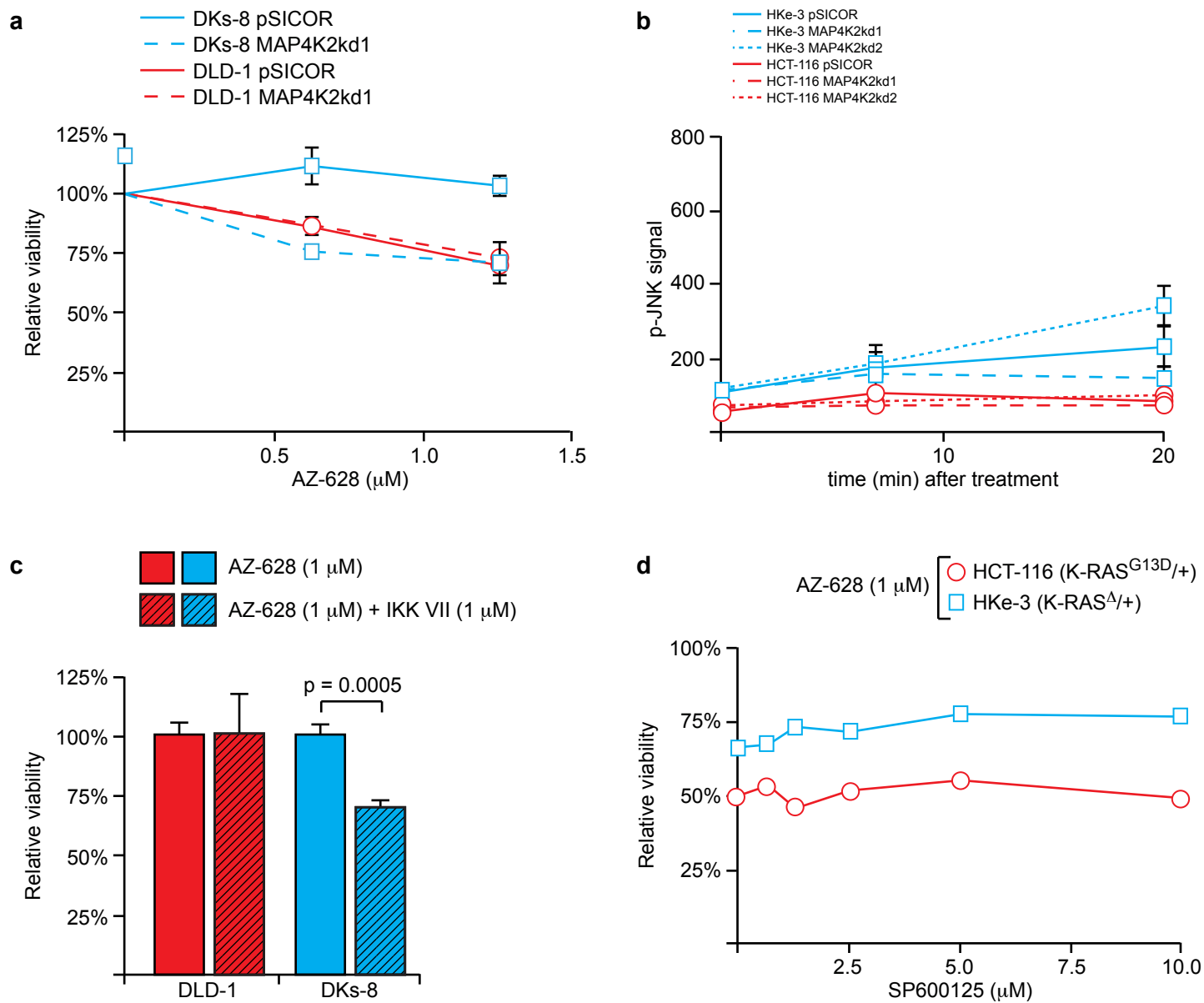

Supplement: Figure S6 — Evaluation of MAP4K2 in the BAY61-3606 response. (a) Cell viability quantified by Syto60 after 72 hours of AZ-628 treatment in DLD-1 or DKs-8 cell lines with MAP4K2 knockdown. Loss of MAP4K2 does not affect AZ-628 response in cells expressing mutant K-RAS, but enhances the effect of AZ-628 in cells expressing wild-type K-RAS. (b) Time course of phospho-JNK (Thr183/Tyr185) after 1 µM AZ-628 treatment in HCT-116 (red lines) or HKe-3 (blue line) cells with MAP4K2 knock down, as measured by Bio-Plex. Relative signal was normalized to a master control lysate. Error bars represent SEM for 3 independent experiments. JNK signaling was enhanced in HKe-3 cells but was independent of MAP4K2. (c) Cell viability quantified by Syto60 after 72 hours of combinatorial treatment with IKK inhibitor VII and 1 µM AZ-628. Relative cell viability was normalized to DMSO vehicle treated control for each cell line. Like BAY61-3606, IKK inhibitor VII enhanced the effect of AZ-628 specifically in K-RAS wild-type cells. (d) Cell viability quantified by Syto60 after 72 hours of combinatorial treatment with the JNK inhibitor SP600125 and 1 µM AZ-628. Relative cell viability was normalized to DMSO vehicle treated control for each cell line. Unlike BAY61-3606, SP600125 did not affect AZ-628 sensitivity in K-RAS wild-type cells. (PDF) [file pone.0041343.s006.pdf]

Figure S7

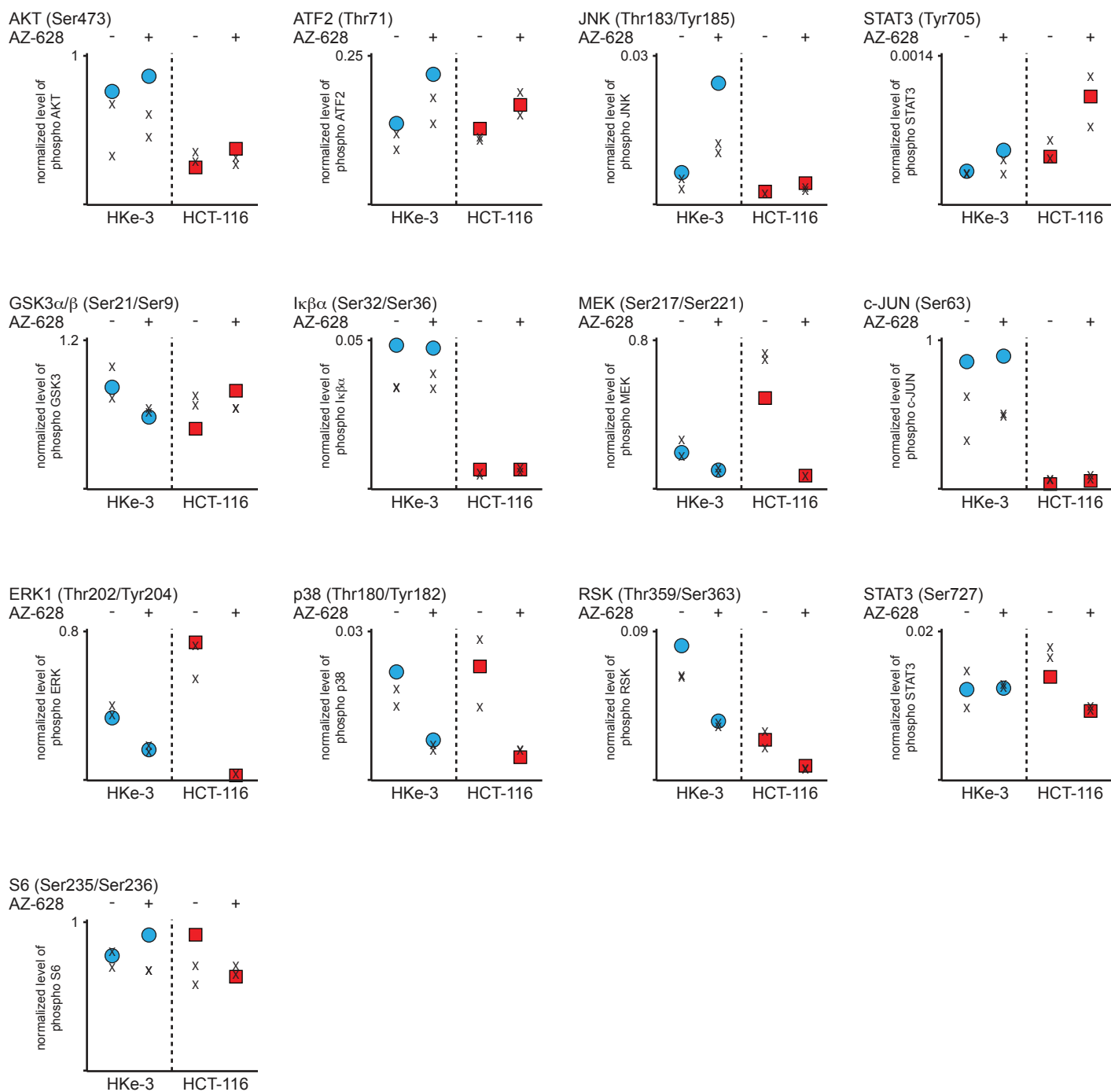

Supplement: Figure S7 — Signaling pathway activity in response to AZ-628. Phospho-protein measurements were made after 45 minutes of exposure to 1 μM AZ-628 or DMSO vehicle control treatment in HCT-116 (red squares) or HKe-3 (blue dots) cells. X's represent measurements from cells with MAP4K2 knock down. All measurements were quantified by Bio-Plex signaling assays. Relative signal was normalized to a master control lysate. (PDF) [file pone.0041343.s007.pdf]
