# Supplementary material for: BAY61-3606 Affects the Viability of Colon Cancer Cells in a Genotype-Directed Manner
Source: PLoS One. 2012 Jul 18;7(7):e41343. doi: 10.1371/journal.pone.0041343 (PMC3399817; doi:10.1371/journal.pone.0041343)
Supplement: Table S1 — Active site binding inhibition data. (PDF) [file pone.0041343.s008.pdf]

Table S1 active site binding inhibition data*

Ambit Gene Symbol	Entrez Gene Symbol	BAY61-3606	BAY derivative 6	BAY derivative 8	BAY derivative 1	BAY derivative 21	BAY derivative 28
AAK1	AAK1	1.6	52	70	45	75	93
ABL1(E255K)-phosphorylated	ABL1	100	60	71	68	93	100
ABL1(F317I)-nonphosphorylated	ABL1	100	100	100	79	100	100
ABL1(F317I)-phosphorylated	ABL1		86	100	100	100	84
ABL1(F317L)-nonphosphorylated	ABL1	95	100	100	100	96	91
ABL1(F317L)-phosphorylated	ABL1		100	100	46	100	100
ABL1(H396P)-nonphosphorylated	ABL1	96	92	100	99	100	93
ABL1(H396P)-phosphorylated	ABL1		100	100	76	100	100
ABL1(M351T)-phosphorylated	ABL1	81	92	100	64	100	100
ABL1(Q252H)-nonphosphorylated	ABL1	85	58	86	66	90	86
ABL2	ABL2	95	93	79	100	100	92
ACVR1	ACVR1	78	98	100	100	100	97
ACVR1B	ACVR1B	100	100	94	100	97	86
ACVR2A	ACVR2A	100	100	100	100	100	100
ACVR2B	ACVR2B	77	100	100	100	98	95
ACVRL1	ACVRL1	100	100	100	100	100	100
ADCK3	CABC1	99	87	95	49	100	100
ADCK4	ADCK4	9.9	69	52	32	100	100
AKT1	AKT1	93	91	86	87	100	100
AKT2	AKT2	79	100	100	100	100	100
AKT3	AKT3	90	100	100	84	100	83
ALK	ALK	84	52	100	71	100	98
AMPK-alpha1	PRKAA1	92	100	100	90	100	97
AMPK-alpha2	PRKAA2	83	100	100	100	100	100
ANKK1	ANKK1	21	100	100	100	100	100
ARK5	NUAK1	62	93	93	100	100	88
ASK1	MAP3K5	100	100	100	100	98	100
ASK2	MAP3K6	81	100	100	100	100	100
AURKA	AURKA	91	87	100	97	100	100
AURKB	AURKB	69	68	88	72	88	84
AURKC	AURKC	92	100	100	100	100	100
AXL	AXL	92	89	97	82	100	91
BIKE	BMP2K	22	53	81	71	100	87
BLK	BLK	100	43	91	66	100	100
BMPR1A	BMPR1A	79	100	100	88	100	100
BMPR1B	BMPR1B	77	100	100	69	100	98
BMPR2	BMPR2	46	87	93	64	94	85
BMX	BMX	95	95	93	85	100	97
BRAF	BRAF	90	100	100	57	100	100
BRAF(V600E)	BRAF	84	100	100	82	100	100
BRK	PTK6	87	59	88	76	100	100
BRSK1	BRSK1	82	100	100	100	100	86
BRSK2	BRSK2	84	100	100	97	100	100
BTK	BTK	50	60	73	45	100	98
CAMK1	CAMK1	62	100	92	97	99	87
CAMK1D	CAMK1D	72	100	100	100	100	93
CAMK1G	CAMK1G	82	98	90	90	90	92
CAMK2A	CAMK2A	64	96	99	100	80	88
CAMK2B	CAMK2B	65	100	95	100	100	90
CAMK2D	CAMK2D	72	100	92	100	79	97
CAMK2G	CAMK2G	78	100	94	95	94	92
CAMK4	CAMK4	100	100	100	100	100	100
CAMKK1	CAMKK1	48	100	98	86	97	98
CAMKK2	CAMKK2	51	100	85	92	87	89
CASK	CASK		100	100	100	100	100
CDC2L1	CDC2L1	95	100	75	97	87	82
CDC2L2	CDC2L2	100	100	80	98	98	97
CDC2L5	CDC2L5		88	100	100	97	99
CDK11	CDC2L6	100	100	100	90	100	100
CDK2	CDK2	86	100	100	84	100	100
CDK3	CDK3	89	94	96	98	100	100
CDK4-cyclinD1	CDK4		100	100	94	100	100
CDK4-cyclinD3	CDK4		95	100	89	95	89
CDK5	CDK5	86	100	100	100	100	100
CDK7	CDK7	33	100	100	68	100	100
CDK8	CDK8	62	100	100	98	100	98
CDK9	CDK9	56	100	80	92	100	92
CDKL1	CDKL1		100	87	100	100	100
CDKL2	CDKL2	78	100	100	98	100	95
CDKL3	CDKL3	100	100	99	92	96	96
CDKL5	CDKL5	60	100	96	100	99	93
CHEK1	CHEK1	100	90	84	100	100	90
CHEK2	CHEK2	100	97	98	95	100	91
CIT	CIT	0.4	95	85	78	90	80
CLK1	CLK1	11	100	88	41	100	100
CLK2	CLK2	12	63	74	80	97	83
CLK3	CLK3	100	100	100	80	100	100

Table S1 (cont.) active site binding inhibition data*

Ambit Gene Symbol	Entrez Gene Symbol	BAY61-3606	BAY derivative 6	BAY derivative 8	BAY derivative 1	BAY derivative 21	BAY derivative 28
CLK4	CLK4	11	100	100	54	100	97
CSF1R	CSF1R	51	42	87	60	100	77
CSK	CSK	68	99	100	100	99	100
CSNK1A1	CSNK1A1	100	100	100	92	99	100
CSNK1A1L	CSNK1A1L		100	100	97	100	100
CSNK1D	CSNK1D	74	100	100	100	100	100
CSNK1E	CSNK1E	94	100	100	82	100	100
CSNK1G1	CSNK1G1	84	94	94	100	97	92
CSNK1G2	CSNK1G2	100	100	100	100	100	82
CSNK1G3	CSNK1G3	81	100	96	94	100	92
CSNK2A1	CSNK2A1	70	86	90	72	97	81
CSNK2A2	CSNK2A2	54	100	100	62	100	100
CTK	MATK	44	98	100	71	100	100
DAPK1	DAPK1	66	76	82	82	74	88
DAPK2	DAPK2	66	100	100	97	100	89
DAPK3	DAPK3	71	100	100	95	100	98
DCAMKL1	DCLK1	93	87	95	67	97	93
DCAMKL2	DCLK2	86	100	89	83	100	94
DCAMKL3	DCLK3	100	100	100	100	100	100
DDR1	DDR1	99	100	100	100	100	90
DDR2	DDR2	74	100	96	82	96	89
DLK	MAP3K12	87	100	100	97	100	100
DMPK	DMPK	72	100	100	100	100	100
DMPK2	CDC42BPG	86	88	91	96	100	93
DRAK1	STK17A	57	100	100	91	100	100
DRAK2	STK17B	15	100	100	100	100	100
DYRK1A	DYRK1A	24	100	91	91	100	100
DYRK1B	DYRK1B	81	57	87	75	100	91
DYRK2	DYRK2	24	56	78	93	100	91
EGFR	EGFR	87	100	100	94	100	90
EGFR(E746-A750del)	EGFR	49	75	99	84	100	70
EGFR(G719C)	EGFR	68	100	93	79	100	82
EGFR(G719S)	EGFR	71	100	100	83	100	84
EGFR(L747-E749del, A750P)	EGFR	83	74	75	64	92	76
EGFR(L747-S752del, P753S)	EGFR	83	76	85	67	90	91
EGFR(L747-T751del,Sins)	EGFR	80	89	95	73	100	78
EGFR(L858R)	EGFR	100	88	92	84	100	96
EGFR(L858R,T790M)	EGFR	76	85	86	63	86	86
EGFR(L861Q)	EGFR	81	68	86	51	100	70
EGFR(S752-I759del)	EGFR	74	72	97	75	100	84
EGFR(T790M)	EGFR		48	60	95	66	66
EIF2AK1	EIF2AK1		88	100	100	100	95
EPHA1	EPHA1	81	81	85	75	100	88
EPHA2	EPHA2	100	89	100	87	100	96
EPHA3	EPHA3	83	100	89	78	83	88
EPHA4	EPHA4	91	92	74	99	93	100
EPHA5	EPHA5	77	100	94	100	100	100
EPHA6	EPHA6	97	100	72	98	95	99
EPHA7	EPHA7	98	100	97	88	100	100
EPHA8	EPHA8	83	100	95	92	100	100
EPHB1	EPHB1	90	100	86	99	100	100
EPHB2	EPHB2	100	99	100	75	93	85
EPHB3	EPHB3	98	84	77	65	100	100
EPHB4	EPHB4	86	97	82	94	100	100
EPHB6	EPHB6	17	56	100	51	100	100
ERBB2	ERBB2	93	71	95	69	80	100
ERBB3	ERBB3	100	87	94	30	93	91
ERBB4	ERBB4	90	100	100	100	100	100
ERK1	MAPK3	100	100	100	100	100	85
ERK2	MAPK1	68	70	70	100	86	91
ERK3	MAPK6	73	88	98	100	100	83
ERK4	MAPK4	87	100	100	100	92	89
ERK5	MAPk7	87	100	100	100	100	100
ERK8	MAPK15	92	98	97	85	85	90
ERN1	ERN1	72	100	100	100	100	100
FAK	PTK2	69	95	89	86	99	100
FER	FER	69	100	100	88	100	100
FES	FES	88	100	80	87	88	95
FGFR1	FGFR1	93	100	100	90	91	87
FGFR2	FGFR2	100	83	94	80	100	89
FGFR3	FGFR3	100	100	100	95	100	93
FGFR3(G697C)	FGFR3	83	70	82	98	99	85
FGFR4	FGFR4	100	100	100	78	100	100
FGR	FGR	80	54	59	54	88	100
FLT1	FLT1	100	100	99	100	100	99
FLT3	FLT3	26	52	97	79	100	100
FLT3(D835H)	FLT3	7.9	42	89	12	100	100

Table S1 (cont.) active site binding inhibition data*

Ambit Gene Symbol	Entrez Gene Symbol	BAY61-3606	BAY derivative 6	BAY derivative 8	BAY derivative 1	BAY derivative 21	BAY derivative 28
FLT3(D835Y)	FLT3	2.5	50	80	11	100	97
FLT3(ITD)	FLT3	2.1	82	100	34	100	100
FLT3(K663Q)	FLT3	25	63	100	85	100	91
FLT3(N841I)	FLT3	1.5	53	74	41	81	77
FLT3(R834Q)	FLT3		100	100	63	100	100
FLT4	FLT4	100	86	99	90	100	91
FRK	FRK	100	84	93	87	100	89
FYN	FYN	100	61	80	72	100	93
GAK	GAK	30	43	45	12	97	89
GCN2(Kin.Dom.2,S808G)	EIF2AK4	54	99	86	68	100	93
GRK1	GRK1	65	100	94	100	100	100
GRK4	GRK4	73	100	100	100	100	100
GRK7	GRK7	59	100	100	91	100	100
GSK3A	GSK3A	17	100	100	93	99	93
GSK3B	GSK3B	39	100	100	100	100	100
HCK	HCK	88	83	97	80	100	95
HIPK1	HIPK1	8.8	85	86	56	99	93
HIPK2	HIPK2	9	100	100	81	96	77
HIPK3	HIPK3	36	95	88	86	100	82
HIPK4	HIPK4	29	98	100	92	100	90
HPK1	MAP4K1	43	100	85	64	100	99
HUNK	HUNK	67	75	80	72	81	95
ICK	ICK	23	100	100	100	100	89
IGF1R	IGF1R	97	79	85	89	96	73
IKK-alpha	CHUK	0.25	100	100	100	100	100
IKK-beta	IKBKB	37	89	86	87	86	73
IKK-epsilon	IKBKE	33	96	86	97	86	99
INSR	INSR	60	99	100	63	100	100
INSRR	INSRR	80	91	86	91	100	96
IRAK1	IRAK1	8	92	99	100	100	100
IRAK3	IRAK3	16	57	70	52	100	84
IRAK4	IRAK4		100	100	82	100	100
ITK	ITK	100	100	90	90	100	100
JAK1(JH1domain-catalytic)	JAK1	71	48	90	75	100	100
JAK1(JH2domain-pseudokinase)	JAK1	80	1.2	66	8.5	100	100
JAK2(JH1domain-catalytic)	JAK2	90	13	85	20	100	97
JAK3(JH1domain-catalytic)	JAK3	100	100	100	100	100	100
JNK1	MAPK8	18	95	91	82	100	100
JNK2	MAPK9	100	100	99	89	81	100
JNK3	MAPK10	41	100	91	96	98	99
KIT	KIT	8.4	60	90	47	100	98
KIT(A829P)	KIT		35	70	63	100	96
KIT(D816H)	KIT		61	84	55	100	100
KIT(D816V)	KIT	4.7	22	48	24	100	82
KIT(L576P)	KIT	0.4	38	94	24	100	100
KIT(V559D)	KIT	3	54	90	46	100	89
KIT(V559D,T670I)	KIT	9.3	87	89	76	91	83
KIT(V559D,V654A)	KIT	71	100	100	100	100	99
LATS1	LATS1	100	100	100	100	100	99
LATS2	LATS2	45	88	85	96	100	99
LCK	LCK	87	41	78	59	100	100
LIMK1	LIMK1	100	91	88	100	95	97
LIMK2	LIMK2	98	89	97	95	100	92
LKB1	STK11	95	72	89	99	75	83
LOK	STK10	83	53	84	52	100	86
LRRK2	LRRK2		95	100	80	98	85
LRRK2(G2019S)	LRRK2		92	97	97	93	94
LTK	LTK	46	30	100	64	99	88
LYN	LYN	100	96	100	65	100	89
LZK	MAP3K13	64	100	100	100	100	100
MAK	MAK	68	100	100	76	100	100
MAP3K1	MAP3K1	100	100	100	100	100	100
MAP3K15	MAP3K15	100	86	96	100	100	84
MAP3K2	MAP3K2	55	88	100	70	100	94
MAP3K3	MAP3K3	97	85	93	100	85	96
MAP3K4	MAP3K4	100	99	75	84	89	85
MAP4K2	MAP4K2	6	100	100	90	100	100
MAP4K3	MAP4K3	97	100	100	100	100	100
MAP4K4	MAP4K4	94	86	90	100	100	80
MAP4K5	MAP4K5	80	100	100	100	100	92
MAPKAPK2	MAPKAPK2	100	93	100	100	100	91
MAPKAPK5	MAPKAPK5	100	100	100	100	97	100
MARK1	MARK1	100	100	100	88	91	100
MARK2	MARK2	63	65	75	54	47	85
MARK3	MARK3	100	100	100	100	95	100
MARK4	MARK4	88	75	100	74	77	100
MAST1	MAST1	52	74	100	87	100	100

Table S1 (cont.) active site binding inhibition data*

Ambit Gene Symbol	Entrez Gene Symbol	BAY61-3606	BAY derivative 6	BAY derivative 8	BAY derivative 1	BAY derivative 21	BAY derivative 28
MEK1	MAP2K1	100	100	100	100	100	100
MEK2	MAP2K2	86	100	100	100	100	100
MEK3	MAP2K3	21	94	100	97	100	100
MEK4	MAP2K4	45	100	100	54	97	100
MEK5	MAP2K5		100	100	79	100	100
MEK6	MAP2K6	40	97	100	81	95	92
MELK	MELK	87	91	92	100	90	94
MERTK	MERTK	78	100	100	100	100	100
MET	MET	100	100	100	99	100	83
MET(M1250T)	MET	63	100	99	64	100	100
MET(Y1235D)	MET	100	100	100	100	100	100
MINK	MINK1	62	100	100	83	100	99
MKK7	MAP2K7		86	89	100	100	83
MKNK1	MKNK1	52	72	89	39	86	82
MKNK2	MKNK2	1.7	100	96	100	100	100
MLCK	MLCK	100	100	100	100	100	98
MLK1	MAP3K9	100	100	100	88	100	77
MLK2	MAP3K10	91	100	100	93	94	94
MLK3	MAP3K11	77	100	100	100	100	100
MRCKA	CDC42BPA	83	79	100	84	99	98
MRCKB	CDC42BPB	99	100	100	100	100	90
MST1	STK4	85	87	95	87	100	100
MST1R	MST1R	74	100	100	100	100	100
MST2	STK3	97	82	92	63	100	90
MST3	STK24	56	100	95	84	100	96
MST4	MST4	57	72	97	100	100	100
MTOR	FRAP1		67	72	100	100	100
MUSK	MUSK	76	100	92	100	100	100
MYLK	MYLK	55	100	100	90	100	100
MYLK2	MYLK2	86	89	95	80	95	82
MYLK4	MYLK4		100	100	79	98	87
MYO3A	MYO3A	80	100	100	100	100	96
MYO3B	MYO3B	72	100	100	99	93	98
NDR1	STK38	80	100	100	91	100	99
NDR2	STK38L	68	93	82	78	81	85
NEK1	NEK1	27	91	91	92	92	89
NEK11	NEK11		84	83	76	100	89
NEK2	NEK2	77	88	90	72	98	89
NEK3	NEK3		100	100	91	100	100
NEK4	NEK4		85	87	93	100	93
NEK5	NEK5	86	72	84	94	100	84
NEK6	NEK6	63	100	97	100	100	100
NEK7	NEK7	60	100	100	95	100	100
NEK9	NEK9	90	100	100	90	100	100
NIM1	MGC42105	88	100	93	93	100	100
NLK	NLK	88	82	86	93	81	85
OSR1	OXSR1	100	100	96	100	100	86
p38-alpha	MAPK14	100	95	92	92	93	96
p38-beta	MAPK11	100	80	80	79	87	79
p38-delta	MAPK13	75	100	87	96	93	73
p38-gamma	MAPK12	47	56	70	76	61	91
PAK1	PAK1	94	83	86	100	98	86
PAK2	PAK2	80	76	85	91	98	84
PAK3	PAK3	92	100	100	100	100	100
PAK4	PAK4	85	90	100	95	100	95
PAK6	PAK6	65	99	98	100	97	100
PAK7	PAK7	72	98	100	100	96	100
PCTK1	PCTK1	86	100	100	100	100	99
PCTK2	PCTK2	100	100	81	100	100	100
PCTK3	PCTK3	92	100	78	80	94	80
PDGFRA	PDGFRA	67	100	92	100	93	100
PDGFRB	PDGFRB	47	67	95	40	100	100
PDPK1	PDPK1	99	100	100	100	100	100
PFCDPK1(P.falciparum)	PFB0815w		67	91	29	100	90
PFPK5(P.falciparum)	MAL13P1.279		100	95	98	100	100
PFTAIRE2	PFTK2	85	100	100	100	100	100
PFTK1	PFTK1	100	100	86	97	100	97
PHKG1	PHKG1	79	100	100	98	100	92
PHKG2	PHKG2	26	100	100	100	100	90
PIK3C2B	PIK3C2B	60	100	100	100	100	98
PIK3C2G	PIK3C2G	23	100	100	98	100	97
PIK3CA	PIK3CA	92	100	100	100	100	100
PIK3CA(C420R)	PIK3CA	100	100	97	100	93	100
PIK3CA(E542K)	PIK3CA	99	100	100	100	100	100
PIK3CA(E545A)	PIK3CA	90	100	100	100	100	100
PIK3CA(E545K)	PIK3CA	100	100	100	100	100	100
PIK3CA(H1047L)	PIK3CA	82	100	100	90	100	100

Table S1 (cont.) active site binding inhibition data*

Ambit Gene Symbol	Entrez Gene Symbol	BAY61-3606	BAY derivative 6	BAY derivative 8	BAY derivative 1	BAY derivative 21	BAY derivative 28
PIK3CA(H1047Y)	PIK3CA	80	100	100	89	100	100
PIK3CA(I800L)	PIK3CA		100	100	70	100	98
PIK3CA(M1043I)	PIK3CA	80	100	100	91	100	96
PIK3CA(Q546K)	PIK3CA	87	100	100	88	100	95
PIK3CB	PIK3CB	92	100	100	100	100	86
PIK3CD	PIK3CD	81	100	100	92	100	100
PIK3CG	PIK3CG	26	100	100	100	100	100
PIK4CB	PI4KB	24	100	100	83	100	99
PIM1	PIM1	78	100	99	100	100	90
PIM2	PIM2	87	91	83	98	98	91
PIM3	PIM3	81	82	90	92	99	85
PIP5K1A	PIP5K1A	64	86	96	78	100	95
PIP5K1C	PIP5K1C		71	77	74	91	80
PIP5K2B	PIP4K2B	100	100	100	96	100	89
PIP5K2C	PIP4K2C		99	100	88	100	94
PKAC-alpha	PRKACA	90	72	100	97	100	100
PKAC-beta	PRKACB	76	95	99	89	100	94
PKMYT1	PKMYT1	88	95	92	88	100	90
PKN1	PKN1	47	100	100	95	100	98
PKN2	PKN2	34	91	100	98	99	100
PKNB(M.tuberculosis)	pknB		99	100	92	100	100
PLK1	PLK1	98	100	100	87	100	100
PLK2	PLK2	50	100	100	92	97	100
PLK3	PLK3	37	100	84	92	98	100
PLK4	PLK4	35	100	100	82	100	100
PRKCD	PRKCD	94	100	100	80	19	85
PRKCE	PRKCE	30	100	100	100	100	100
PRKCH	PRKCH	58	100	100	93	100	100
PRKCI	PRKCI		100	77	100	100	100
PRKCQ	PRKCQ	88	100	100	100	100	100
PRKD1	PRKD1	39	100	100	100	100	100
PRKD2	PRKD2	53	100	100	100	100	93
PRKD3	PRKD3	37	100	100	100	100	90
PRKG1	PRKG1	70	100	100	100	100	96
PRKG2	PRKG2	70	100	88	88	100	99
PRKR	EIF2AK2	27	100	98	100	100	10
PRKX	PRKX	75	73	83	68	100	68
PRP4	PRPF4B	77	100	86	92	100	100
PYK2	PTK2B	94	100	100	100	100	100
QSK	KIAA0999	100	87	87	78	83	91
RAF1	RAF1	81	100	100	92	100	100
RET	RET	98	100	100	94	100	100
RET(M918T)	RET	93	72	100	86	100	100
RET(V804L)	RET	95	95	100	88	100	100
RET(V804M)	RET	85	94	100	100	89	74
RIOK1	RIOK1	31	95	95	67	100	97
RIOK2	RIOK2	4	80	79	52	92	86
RIOK3	RIOK3	98	80	100	66	92	90
RIPK1	RIPK1	86	100	100	95	100	100
RIPK2	RIPK2	70	100	100	97	100	98
RIPK4	RIPK4	19	93	90	85	95	82
RIPK5	DSTKY		100	90	59	100	100
ROCK1	ROCK1	3.2	81	97	83	96	89
ROCK2	ROCK2	13	96	100	99	99	88
ROS1	ROS1	45	36	80	43	100	99
RPS6KA1(Kin.Dom.1-N-terminal)	RPS6KA1	96	82	93	89	89	92
RPS6KA1(Kin.Dom.2-C-terminal)	RPS6KA1	50	100	97	86	100	98
RPS6KA2(Kin.Dom.1-N-terminal)	RPS6KA2	92	96	100	100	90	86
RPS6KA2(Kin.Dom.2-C-terminal)	RPS6KA2	89	100	92	96	87	97
RPS6KA3(Kin.Dom.1-N-terminal)	RPS6KA3	66	96	94	81	94	76
RPS6KA4(Kin.Dom.1-N-terminal)	RPS6KA4	32	74	81	59	88	92
RPS6KA4(Kin.Dom.2-C-terminal)	RPS6KA4	60	86	100	100	100	100
RPS6KA5(Kin.Dom.1-N-terminal)	RPS6KA5	90	89	100	100	100	100
RPS6KA5(Kin.Dom.2-C-terminal)	RPS6KA5	89	97	93	97	100	100
RPS6KA6(Kin.Dom.1-N-terminal)	RPS6KA6	95	70	100	75	100	94
RPS6KA6(Kin.Dom.2-C-terminal)	RPS6KA6	79	93	81	30	100	87
S6K1	RPS6KB1		84	87	96	100	89
SBK1	SBK1	96	100	92	90	90	100
SgK110	SgK110	100	100	100	93	100	100
SGK3	SGK3		100	100	100	100	100
SIK	SNF1LK	80	100	92	99	100	100
SIK2	SNF1LK2	100	64	98	74	100	94
SLK	SLK	58	28	74	39	100	100
SNARK	NUAK2	32	89	96	88	100	100
SNRK	SNRK		94	100	100	100	87
SRC	SRC	79	51	59	50	100	100
SRMS	SRMS	65	97	100	75	100	98

Table S1 (cont.) active site binding inhibition data*

Ambit Gene Symbol	Entrez Gene Symbol	BAY61-3606	BAY derivative 6	BAY derivative 8	BAY derivative 1	BAY derivative 21	BAY derivative 28
SRPK1	SRPK1	88	75	100	79	100	100
SRPK2	SRPK2	53	90	100	86	100	100
SRPK3	SRPK3	74	100	100	100	100	100
STK16	STK16	30	47	58	68	68	83
STK33	STK33	87	100	100	96	100	99
STK35	STK35	88	91	92	80	96	96
STK36	STK36	74	100	100	88	100	88
STK39	STK39	46	97	100	99	100	91
SYK	SYK	6.1	48	30	35	100	100
TAK1	MAP3K7	63	96	92	71	99	100
TAO1	TAOK2	100	96	100	75	100	94
TAOK1	TAOK1	95	100	100	52	100	85
TAOK3	TAOK3	100	100	100	81	100	100
TBK1	TBK1	47	100	78	100	86	97
TEC	TEC	100	100	92	100	100	98
TESK1	TESK1	90	100	88	100	100	97
TGFBR1	TGFBR1	99	99	100	100	100	88
TGFBR2	TGFBR2	31	100	100	90	100	100
TIE1	TIE1	100	94	100	85	100	97
TIE2	TEK	100	100	96	85	100	87
TLK1	TLK1	89	57	87	74	77	80
TLK2	TLK2	81	100	100	92	100	91
TNIK	TNIK	69	98	100	92	99	100
TNK1	TNK1	82	56	87	86	100	98
TNK2	TNK2	96	53	94	100	99	95
TNNI3K	TNNI3K	100	100	95	96	100	98
TRKA	NTRK1	71	98	100	73	100	92
TRKB	NTRK2	83	74	96	73	100	100
TRKC	NTRK3	92	98	100	82	100	97
TRPM6	TRPM6		95	96	66	97	100
TSSK1B	TSSK1B	96	100	98	100	100	100
TTK	TTK	51	74	100	72	100	100
TXK	TXK	95	78	87	86	100	92
TYK2(JH1domain-catalytic)	TYK2	99	25	85	48	94	93
TYK2(JH2domain-pseudokinase)	TYK2	9.2	37	100	7.9	100	100
TYRO3	TYRO3	94	67	86	100	99	98
ULK1	ULK1	97	100	100	85	100	96
ULK2	ULK2	69	86	93	76	97	92
ULK3	ULK3	51	100	97	86	100	100
VEGFR2	KDR	94	100	100	100	100	91
VRK2	VRK2		88	99	100	100	100
WEE1	WEE1	89	100	100	100	100	100
WEE2	WEE1B	100	100	100	62	100	100
YANK1	STK32A		100	72	100	89	100
YANK2	STK32B	86	100	100	93	100	96
YANK3	STK32C	53	100	100	100	100	100
YES	YES1	96	57	78	79	81	85
YSK1	STK25	53	82	100	80	73	78
YSK4	YSK4	1.8	60	67	55	82	86
ZAK	ZAK	94	92	78	89	90	86
ZAP70	ZAP70	9	100	100	100	100	100

* Compounds were screened at 1 uM by Ambit KINOMEscan™. Value represent % binding compared to no inhibitor control.
