# Supplementary material for: BAY61-3606 Affects the Viability of Colon Cancer Cells in a Genotype-Directed Manner
Source: PLoS One. 2012 Jul 18;7(7):e41343. doi: 10.1371/journal.pone.0041343 (PMC3399817; doi:10.1371/journal.pone.0041343)
Supplement: Table S2 — shRNAs used in this study. (PDF) [file pone.0041343.s009.pdf]

Table S2 shRNAs used in this study

| Kinase | Gene name | Target* | Target sequence |
|---|---|---|---|
| CDK9 | cyclin-dependent kinase 9 (CDC2-related kinase) | 3UTR | GCACAGTTTGGTCCGTTAGAA |
| CDK9 | cyclin-dependent kinase 9 (CDC2-related kinase) | CDS | AGGGACATGAAGGCTGCTAAT |
| GSK3A | glycogen synthase kinase 3 alpha | CDS | GAGTTCAAGTTCCCTCAGATT |
| GSK3B | glycogen synthase kinase 3 beta | CDS | CCGATTGCGTTATTTCTTCTA |
| GSK3B | glycogen synthase kinase 3 beta | CDS | CCCAAACTACACAGAATTTAA |
| HIPK1 | homeodomain interacting protein kinase 1 | CDS | CAGCCCTTATTCCACTGATAC |
| HIPK1 | homeodomain interacting protein kinase 1 | CDS | CCTTCTGCAGTGCGAAGAAAC |
| HIPK2 | homeodomain interacting protein kinase 2 | 3UTR | AGGGATTAAAGAGGGTGGGAA |
| MAP4K2 | mitogen-activated protein kinase kinase kinase kinase 2 | 3UTR | GAACTATATTGGTATTAAA |
| MAP4K2 | mitogen-activated protein kinase kinase kinase kinase 2 | CDS | GGAGATCACAGATGAAAC |
| NEK1 | NIMA (never in mitosis gene a)-related kinase 1 | 3UTR | CCTCAACTATATGAAAGCATT |
| RIOK1 | RIO kinase 1 (yeast) | CDS | GCAGATATGTATCGCATCAAA |
| RIOK1 | RIO kinase 1 (yeast) | CDS | CCAGACTGTTACAGGATTGAA |
| ROCK1 | Rho-associated, coiled-coil containing protein kinase 1 | CDS | GAGGTAAATGAACACAAAGTA |
| ROCK1 | Rho-associated, coiled-coil containing protein kinase 1 | CDS | GCACCAGTTGTACCCGATTTA |
| ROCK1 | Rho-associated, coiled-coil containing protein kinase 1 | CDS | CGGTTAGAACAAGAGGTAAAT |
| ROCK2 | Rho-associated, coiled-coil containing protein kinase 2 | 3UTR | GCTATGAAGCTTCTTAGTAAG |
| SYK | spleen tyrosine kinase | 3UTR | GTACCTCTTGAAAGTTAA |
| SYK | spleen tyrosine kinase | 3UTR | GCCAGCAGATATAAATAA |

* Target refers to the region of the mRNA that is targeted by the shRNA
